# Loss of tetherin antagonism by Nef impairs SIV replication during acute infection of rhesus macaques

**DOI:** 10.1371/journal.ppat.1008487

**Published:** 2020-04-17

**Authors:** Aidin Tavakoli-Tameh, Sanath Kumar Janaka, Katie Zarbock, Shelby O’Connor, Kristin Crosno, Saverio Capuano, Hajime Uno, Jeffrey D. Lifson, David T. Evans

**Affiliations:** 1 Department of Pathology and Laboratory Medicine, University of Wisconsin-Madison, Madison, Wisconsin, United States of America; 2 Wisconsin National Primate Research Center, University of Wisconsin-Madison, Madison, Wisconsin, United States of America; 3 Department of Biostatistics and Computational Biology, Dana-Farber Cancer Institute, Boston, Massachusetts, United States of America; 4 AIDS and Cancer Virus Program, Leidos Biomedical Research Inc., Frederick National Laboratory for Cancer Research, Frederick, Maryland, United States of America; King's College London, UNITED KINGDOM

## Abstract

Most simian immunodeficiency viruses use Nef to counteract the tetherin proteins of their nonhuman primate hosts. Nef also downmodulates cell-surface CD4 and MHC class I (MHC I) molecules and enhances viral infectivity by counteracting SERINC5. We previously demonstrated that tetherin antagonism by SIV Nef is genetically separable from CD4- and MHC I-downmodulation. Here we show that disruption of tetherin antagonism by Nef impairs virus replication during acute SIV infection of rhesus macaques. A combination of mutations was introduced into the SIV_mac_239 genome resulting in three amino acid substitutions in Nef that impair tetherin antagonism, but not CD3-, CD4- or MHC I-downmodulation. Further characterization of this mutant (SIV_mac_239_AAA_) revealed that these changes also result in partial sensitivity to SERINC5. Separate groups of four rhesus macaques were infected with either wild-type SIV_mac_239 or SIV_mac_239_AAA_, and viral RNA loads in plasma and sequence changes in the viral genome were monitored. Viral loads were significantly lower during acute infection in animals infected with SIV_mac_239_AAA_ than in animals infected with wild-type SIV_mac_239. Sequence analysis of the virus population in plasma confirmed that the substitutions in Nef were retained during acute infection; however, changes were observed by week 24 post-infection that fully restored anti-tetherin activity and partially restored anti-SERINC5 activity. These observations reveal overlap in the residues of SIV Nef required for counteracting tetherin and SERINC5 and selective pressure to overcome these restriction factors *in vivo*.

## Author summary

The SIV Nef protein enhances virus replication by removing certain cellular proteins from the surface of infected cells. Whereas the downmodulation of tetherin enhances virus release from infected cells, the downmodulation of SERINC5, and to a lesser extent SERINC3, increases viral infectivity. Although these Nef-mediated effects on the host cell have been demonstrated *in vitro*, the relevance of these activities to virus replication in primates has not been directly assessed. We therefore introduced mutations into SIV that render Nef ineffective against tetherin and SERINC5. Rhesus macaques infected with this virus exhibited lower acute viremia compared to animals infected with wild-type SIV. We also observed a partial recovery of these functions as a result of the accumulation of compensatory mutations in Nef. This study therefore demonstrates that overcoming restriction by tetherin and SERINC5 is important for efficient replication of immunodeficiency viruses in their infected hosts.

## Introduction

Mammals have evolved a number of restriction factors that interfere with specific stages of virus replication [1]. One such factor, tetherin (BST-2 or CD317), is an interferon inducible transmembrane protein with an unusual topology that includes an N-terminal cytoplasmic domain followed by a membrane spanning domain, an extracellular coiled-coil domain and a C-terminal glycosylphosphatidylinositol anchor [2]. This unique topology enables opposite ends of tetherin homodimers to be incorporated into viral and cellular membranes, thereby linking budding virions to the cell surface [3–7]. In addition to impairing virus release, the antiviral activity of tetherin may be amplified *in vivo* by triggering the production of proinflammatory cytokines as a downstream consequence of NF-κB signaling and by increasing the sensitivity of infected cells to elimination by antibody-dependent cellular cytotoxicity (ADCC) [8–10].

Tetherin impairs the detachment of diverse families of enveloped viruses from infected cells [3–5]. In the case of the primate lentiviruses, most simian immunodeficiency viruses (SIVs) use Nef to counteract the tetherin proteins of their non-human primate hosts [11–13]; however, due to the absence of a five-amino acid sequence in the cytoplasmic domain of human tetherin required for susceptibility to Nef, HIV-1 and HIV-2 instead use their Vpu and Env proteins, respectively, to counteract human tetherin [6, 7, 14–16]. These observations are further supported by studies in animal models indicating that SIV and HIV-1 are under strong selective pressure to maintain mechanisms to counteract tetherin. These include studies revealing compensatory changes in the gp41 cytoplasmic domain of a *nef-*deleted strain of SIV that enable tetherin antagonism by Env [17, 18], changes in HIV-1 during replication in chimpanzees that restore tetherin antagonism by Nef [19], and changes in Vpu of simian-tropic HIV-1 during replication in pig-tailed macaques that confer resistance to macaque tetherin [20].

In addition to tetherin antagonism, SIV Nef has other functional activities that promote virus replication [21–23]. Most of these functions, including CD4 and MHC class I downmodulation, infectivity enhancement and NF-κB activation, are shared by HIV-1 Nef. CD4 downmodulation prevents the exposure of CD4-inducible epitopes in Env that render virus-infected cells susceptible to elimination by ADCC [24–26] and MHC class I downmodulation reduces the susceptibility of infected cells to elimination by virus-specific CD8^+^ T cells [27–29]. The Nef proteins of HIV-1 and SIV also enhance virus infectivity by preventing the incorporation of the multipass transmembrane protein serine incorporator 5 (SERINC5) into virions [30, 31] and stimulate viral gene transcription from the LTR promoter by amplifying NF-κB signaling [32]. Another function of SIV Nef that appears to have been lost by HIV-1 is CD3 downmodulation [33, 34], which was recently shown to contribute to immune evasion by dampening immune activation in SIV-infected animals [35]

We previously demonstrated that the anti-tetherin activity of SIV Nef is genetically separable from CD4- and MHC class I-downregulation, functions of Nef that involve distinct protein surfaces and cellular pathways [15]. In the present study, we engineered an infectious molecular clone of SIV with mutations in Nef that selectively disrupt tetherin antagonism (SIV_mac_239_AAA_). *In vitro* characterization of this virus confirmed that Nef retained CD3-, CD4- and MHC class I-downmodulation, but was unable to fully counteract the effects of SERINC5 on viral infectivity. Rhesus macaques infected with SIV_mac_239_AAA_ exhibited a significant reduction in acute viremia compared to animals infected with wild-type SIV_mac_239 and the accumulation of genetic changes in *nef* that restore resistance to tetherin and SERINC5.

## Results

### Amino acid changes in Nef that selectively disrupt tetherin antagonism

**Fig 1:**
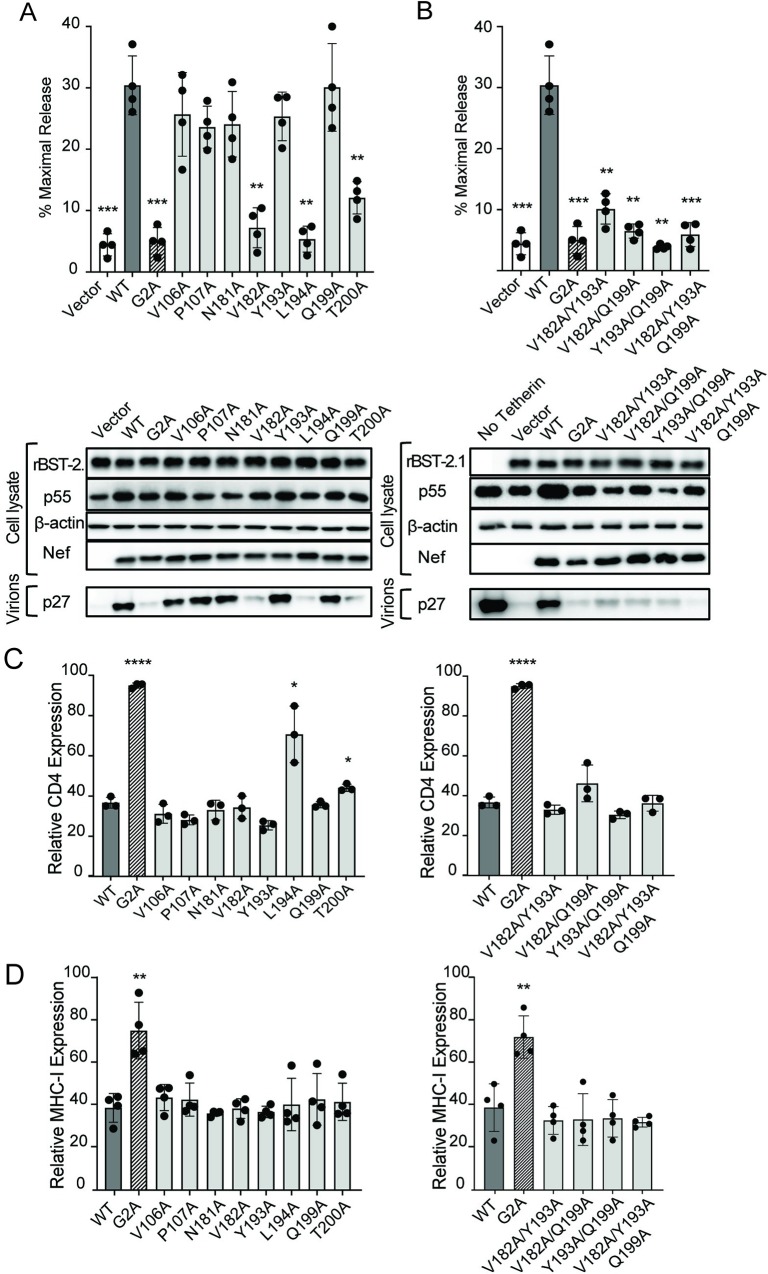
Amino acid changes in Nef that disrupt tetherin antagonism, but not CD4 or MHC class I downregulation. SIV Nef mutants with individual (A) or combinations of double or triple (B) alanine substitutions were tested for the ability to rescue the release of *nef*-deleted SIV in the presence of rhesus tetherin. 293T cells were co-transfected with SIV_mac_239Δ*nef* together with constructs expressing rhesus macaque tetherin and the indicated SIV Nef mutants. Percent maximal virus release was calculated from the accumulation of SIV p27 in the culture supernatant of cells transfected with tetherin relative to cells transfected with empty vector. Controls include virus release in the absence of Nef (Vector), or in the presence of wild-type Nef (WT) or a Nef mutant with a glycine-to-alanine substitution at position 2 (G2A). Differences in virus release were corroborated by western blot analysis. Virion and cell lysate proteins were separated by SDS-PAGE, transferred to PVDF membranes and stained with antibodies to tetherin (BST-2.1), β-actin, the SIV Gag (p55 & p27) and Nef proteins. The SIV Nef mutants were also tested for the ability to downmodulate CD4 (C) and MHC class I (D) molecules. TZM-bl cells and Jurkat cells were transfected with bicistronic constructs that express GFP and either WT Nef (black), Nef G2A (striped) or the indicated Nef mutants (grey). At 48 hours post-transfection, relative levels of CD4 and MHC class I expression were determined by comparing the mean fluorescence intensities of staining on GFP^+^ cells expressing Nef to GFP^+^ cells transfected with an empty vector (white). Error bars indicate standard deviation of the mean for at least three independent experiments and significant differences with respect to WT Nef are indicated by asterisks (*p<0.05, ** *p*<0.01, *** *p*<0.001 & **** *p*<0.0001, two-tailed unpaired *t*-test with Welch’s correction in case of unequal variance).

We previously identified four pairs of alanine substitutions in the SIV_mac_239 Nef protein that impair tetherin antagonism without affecting CD4 or MHC class I downregulation [15]. To further define the residues required for counteracting tetherin, Nef mutants with individual substitutions at these positions were tested for anti-tetherin activity in virus release assays, and for CD4- and MHC class I-downregulation, which involve distinct protein surfaces and cellular pathways [24, 25, 29]. These mutants were compared to wild-type Nef and to a Nef mutant with a glycine-to-alanine substitution at position 2 (G2A) that broadly impairs Nef function by preventing myristoylation and localization of the protein to cellular membranes [12]. Whereas individual alanine substitutions at positions 106 and 107 were not sufficient to disrupt tetherin antagonism, substitutions at positions 182, 194 and 200 (V182A, L194A and T200A) significantly reduced virus release compared to wild-type Nef (Fig 1A); however, since two of these substitutions (L194A and T200A) also impaired CD4 downregulation (Fig 1C), combinations of double mutations were also tested. Although substitutions at positions 193 and 199 did not impair tetherin antagonism individually (Fig 1A), a combination of substitutions at both positions (Y193A/Q199A) dramatically reduced virus release (Fig 1B). Moreover, these changes could be combined, either separately or together, with the V182A substitution without affecting CD4 or MHC class I downmodulation (Fig 1C and 1D & S1 Fig). Hence, these experiments identify a combination of three alanine substitutions in the flexible loop of the SIV_mac_239 Nef protein (V182A, Y193A and Q199A) that disrupt tetherin antagonism, but not CD4 or MHC class I downregulation.

### An infectious molecular clone of SIV with Nef substitutions that selectively impair tetherin antagonism

To create an infectious molecular clone of SIV that is susceptible to tetherin, but retains other Nef functions, nucleotide changes encoding the V182A, Y193A and Q199A substitutions were introduced into the SIV_mac_239 genome. For the Y193A and Q199A changes, it was possible to select codons that require two nucleotide changes to restore the wild-type residue, making it more difficult for the virus to revert to the wild-type sequence during replication in animals. Since this region of *nef* overlaps with the U3 region of the LTR, these mutations were introduced into both LTRs to prevent the recovery of wild-type virus as result of recombination. Thus, the final virus construct (SIV_mac_239_AAA_) contains a combination of five nucleotide changes in both LTRs that introduce V182A, Y193A and Q199A substitutions in the flexible loop region of Nef.

**Fig 2:**
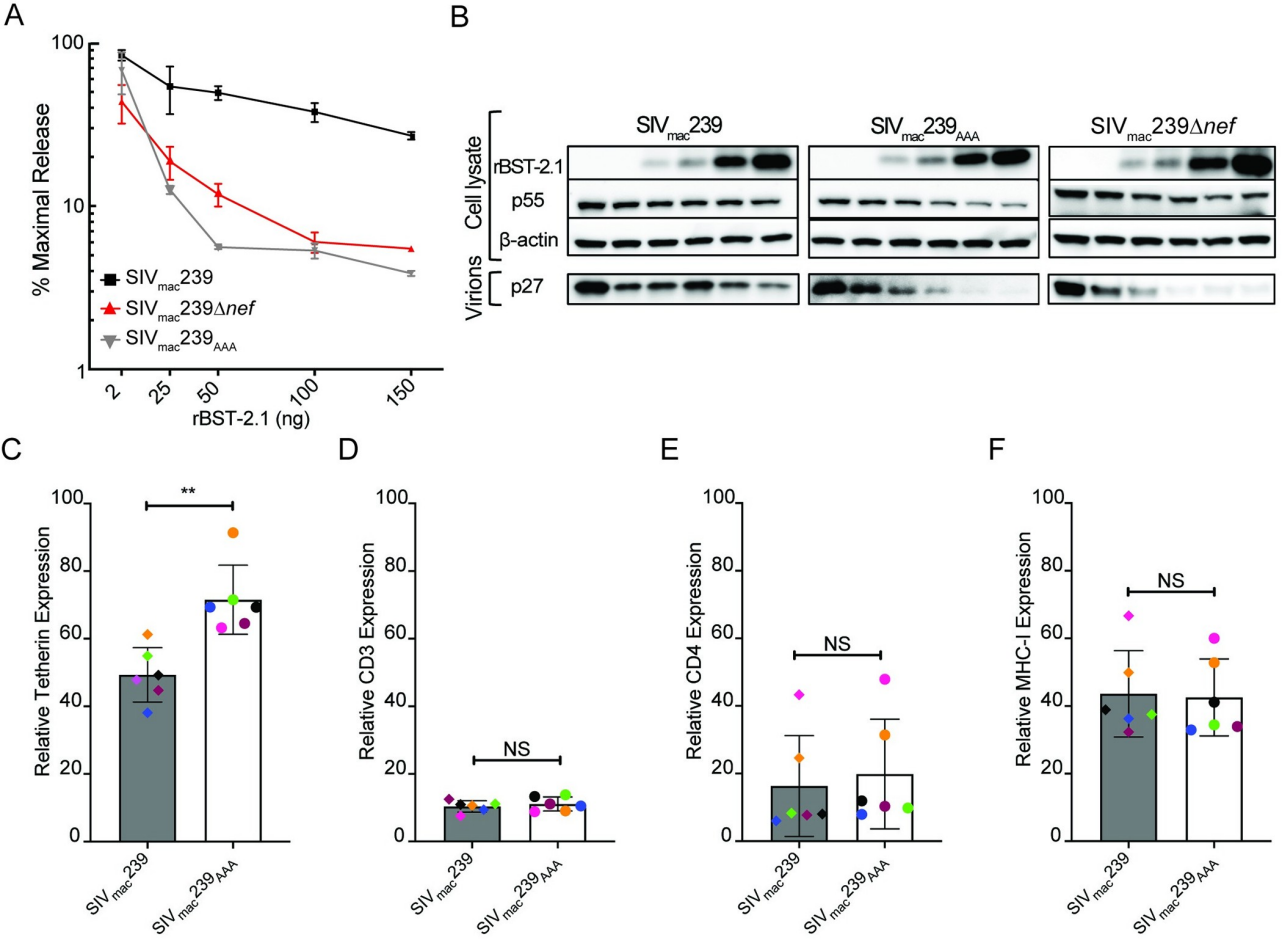
Phenotypic characterization an infectious molecular clone of SIV with substitutions in Nef that selectively impair tetherin antagonism. SIV_mac_239_AAA_, SIV_mac_239 and SIV_mac_239Δ*nef* were tested for resistance to tetherin (A & B), and for downmodulation of tetherin (C), CD3 (D), CD4 (E) and MHC I (F) from the surface of infected primary CD4^+^ lymphocytes. (A) 293T cells were co-transfected with full-length clones for SIV_mac_239, SIV_mac_239Δ*nef* or SIV_mac_239_AAA_ and increasing amounts of an expression construct for rhesus tetherin (rBST-2.1). The accumulation of SIV p27 in supernatant was measured by antigen-capture ELISA and percent maximal release was calculated relative to control transfections in the absence of tetherin. (B) Differences in virus release were corroborated by western blot analysis of virions and cell lysates. Proteins were separated by SDS-PAGE, transferred to PVDF membranes and stained with antibodies to tetherin (rBST-2.1), β-actin, and the SIV Gag p27 and p55 proteins. (C-F) Activated CD4^+^ lymphocytes from six different macaques were infected with SIV_mac_239_AAA_, SIV_mac_239 and SIV_mac_239Δ*nef*. On day 6 post-infection, the cells were stained for surface expression of tetherin, CD3, CD4 and MHC I, and for intracellular expression of the SIV Gag protein. Relative expression levels of tetherin (C), CD3 (D), CD4 (E) and MHC I (F) were determined by comparing the gMFI of each of these molecules on virus-infected (CD4^lo^Gag^+^) cells to uninfected cells (CD4^hi^Gag^-^). Error bars indicate standard deviation of the mean and significant differences are indicated by asterisks (**p<0.01 & NS, not significant, two-tailed unpaired t-test with Welch’s correction in case of unequal variance).

In the presence of increasing expression levels of rhesus tetherin, virus release for SIV_mac_239_AAA_ was suppressed to a similar extent as SIV_mac_239Δ*nef* (Fig 2A & 2B). Accordingly, levels of tetherin on the surface of primary rhesus macaque CD4^+^ lymphocytes infected with SIV_mac_239_AAA_ were significantly lower than lymphocytes infected with SIV_mac_239Δ*nef* (Fig 2C). The downmodulation of CD3, CD4 and MHC class I molecules from the surface of infected macaque lymphocytes was also compared. These surface proteins afford a broad assessment of Nef functions, since their downregulation is dependent on different surfaces of Nef, and on AP-1- (MHC I) versus AP-2- (CD3 & CD4) dependent pathways [29, 36–38]. In contrast to tetherin, CD3, CD4 and MHC class I molecules were downmodulated from the surface of cells infected with SIV_mac_239_AAA_ as efficiently as cells infected with wild-type SIV_mac_239 (Fig 2D–2F & S2 Fig). These results confirm impairment of tetherin antagonism for SIV_mac_239_AAA_ without significant loss of CD3-, CD4- or MHC class I-downregulation.

### Infectivity of SIV_mac_239_AAA_ produced in JTAg cells and primary CD4^+^ T cells

**Fig 3:**
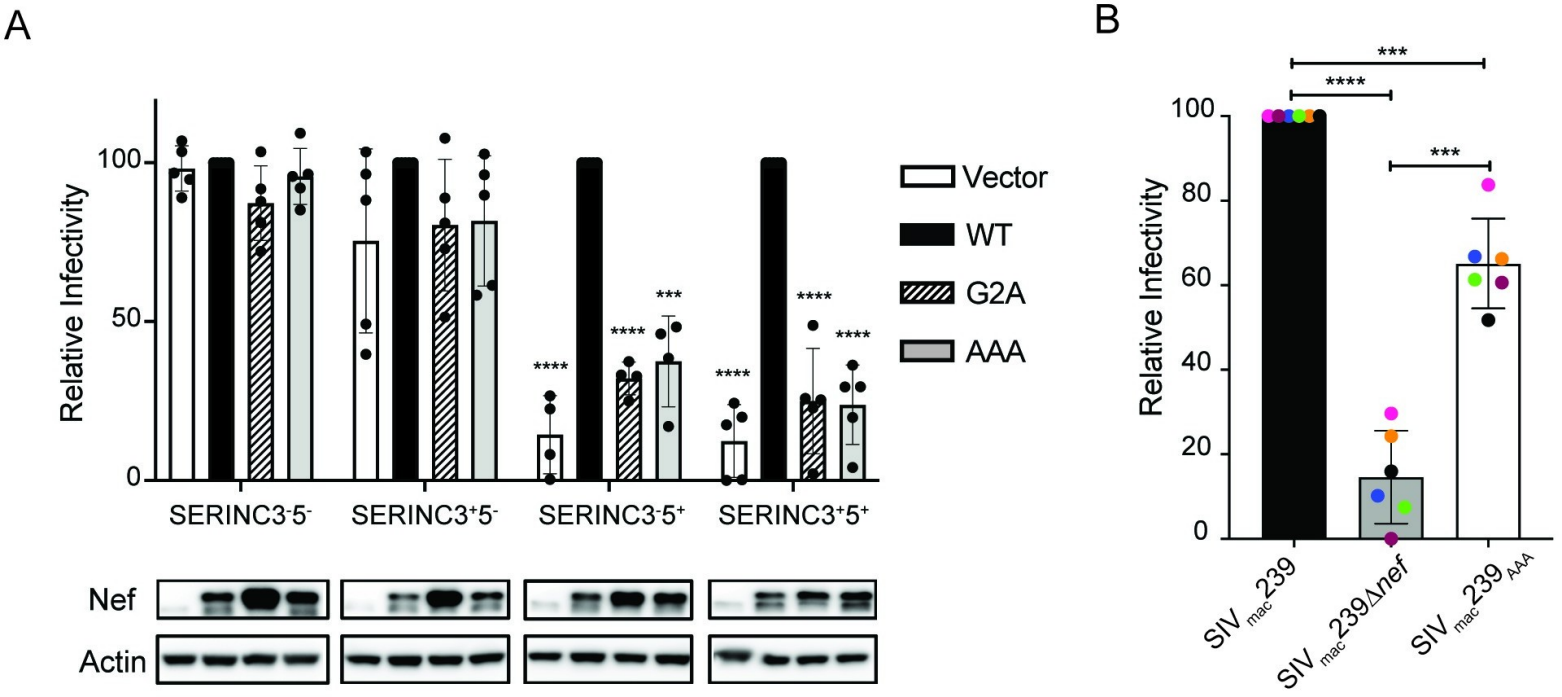
Nef_AAA_ substitutions impair SERINC5 antagonism and SIV_mac_239_AAA_ infectivity. The infectivity of Nef-*trans*-complemented SIV_mac_239Δ*nef* produced in JTAg cells with knock-out mutations in *SERINC3* (SERINC3^-^5^+^), *SERINC5* (SERINC3^+^5^-^), both (SERINC3^-^5^-^) or neither (SERINC3^+^5^+^) (A) and viruses harvested from infected rhesus macaque CD4^+^ lymphocytes (B) were compared. (A) Nef-*trans*-complemented virus was collected 48-hours after co-transfection of JTAg cells with SIV_mac_239Δ*nef* and expression constructs for wild-type Nef (WT), Nef _G2A_ (G2A), Nef_AAA_ (AAA) or empty vector (Vector). Nef expression was also verified by western blot analysis of JTAg cell lysates. (B) SIV_mac_239, SIV_mac_239Δ*nef* and SIV_mac_239_AAA_ were collected after six days of replication in activated CD4^+^ lymphocytes from six different rhesus macaques, virus concentrations were measured by SIV p27 antigen-capture ELISA, and C8166-SEAP cells were infected in triplicate with equivalent doses of each virus (0.5 ng p27 per 1x10^4^ cells). Secreted-alkaline phosphatase (SEAP) activity was measured in the cell culture supernatant 72-hours later. Relative infectivity is shown as a percentage of wild-type Nef or SIV_mac_239 infection. Error bars indicate standard deviation of the mean for at least four independent experiments (A) or six different animals (B). Significant differences are indicated by asterisks (*** *p*<0.001 & **** *p*<0.0001, two-tailed unpaired *t*-test with Welch’s correction in case of unequal variance).

Nef also enhances HIV-1 and SIV infectivity by preventing the incorporation of SERINC5 and to a lesser extent SERINC3 into virions [30, 31]. We therefore compared wild-type Nef, Nef_G2A_ and Nef_AAA_ for the ability to rescue the infectivity of SIV_mac_239Δ*nef* produced in JTAg cells with knockout mutations in *SERINC3* (SERINC3^-^5^+^), *SERINC5* (SERINC3^+^5^-^) or both (SERINC3^-^5^-^) [30]. As expected, SERINC5 significantly inhibited the infectivity of SIV_mac_239Δ*nef* prepared without Nef (vector) in SERINC3^+^5^+^ or SERINC3^-^5^+^ JTAg cells and virus infectivity was fully restored by *trans*-complementing with wild-type Nef (Fig 3A). However, Nef_AAA_ was unable to counteract SERINC5. The infectivity of SIV_mac_239Δ*nef* produced in SERINC3^+^5^+^ or SERINC3^-^5^+^ cells in the presence of Nef_AAA_ was similar to virus *trans*-complemented with Nef_G2A_ or empty vector (Fig 3A). In accordance with a much weaker effect of SERINC3, the infectivity of SIV_mac_239Δ*nef* produced in SERINC3^+^5^-^ cells without Nef, or in the presence of either Nef_G2A_ or Nef_AAA_, was lower than virus produced in the presence of wild-type Nef; however, these differences were not significant (Fig 3A).

These results indicate that Nef_AAA_ is deficient for SERINC5 antagonism. However, since JTAg cells express human SERINC5, we also compared the ability of each of these Nefs to rescue the infectivity of SIV_mac_239Δ*nef* produced in 293T cell lines that express human or rhesus macaque SERINC5. Consistent with minimal differences between human and macaque SERINC5 (99% amino acid sequence identity) and evidence that diverse Nef alleles of HIV-1 and SIV do not exhibit species-specificity in counteracting this factor [39], the infectivity of Nef_AAA_-*trans*-complemented virus collected from cells expressing human or rhesus macaque SERINC5 did not differ (S3 Fig).

Similar comparisons of viruses produced in primary rhesus macaque CD4^+^ lymphocytes revealed partial impairment of SIV_mac_239_AAA_ infectivity. SIV_mac_239, SIV_mac_239Δ*nef* and SIV_mac_239_AAA_ were collected in the supernatant of infected CD4^+^ T cells, SIV p27 concentrations were measured by antigen-capture ELISA and C8166-SEAP cells were infected with an equivalent dose of each virus. SIV_mac_239_AAA_ retained >60% of the infectivity of wild-type SIV_mac_239, whereas the infectivity of SIV_mac_239Δ*nef* was reduced to ~15% of wild-type SIV (Fig 3B). The infectivity of SIV_mac_239_AAA_ was both significantly lower than SIV_mac_239 and significantly higher than SIV_mac_239Δ*nef* (Fig 3B). The intermediate infectivity of SIV_mac_239_AAA_ in primary CD4^+^ lymphocytes is therefore consistent with a partial loss of resistance to SERINC5. It is unclear why Nef_AAA_ was more effective at restoring infectivity in primary CD4^+^ T cells than in JTAg cells, since the similar abundance of *SERINC5* transcripts in these cells suggests comparable levels of protein expression (S4 Fig). Differences in the timing, subcellular distribution or relative abundance of Nef when expressed in *cis* from the viral genome in SIV-infected CD4^+^ T cells and in *trans* from a plasmid construct in transfected cells may have resulted in differences in the efficiency of counteracting SERINC5. Nevertheless, the incomplete loss of SIV_mac_239_AAA_ infectivity when produced under physiological conditions in primary CD4^+^ T cells suggest that Nef_AAA_ retains some residual activity against SERINC5.

Additional functions of Nef were also assessed to determine if they were affected by the substitutions in SIV_mac_239_AAA_. These included the ability to counteract a recently reported block to the infectivity of HIV-1 produced in MOLT-3 cells [40] and the potentiation of NF-κB activation [41]. Similar to HIV-1, deletion of *nef* significantly reduced the infectivity of SIV produced in MOLT-3-CCR5 cells (S5A Fig). This phenotype is independent of SERINC5, since SIV_mac_239Δ*nef* exhibited a similar loss of infectivity relative to SIV_mac_239 when produced in MOLT-3-CCR5 cells with knockout mutations in *SERINC5* or both *SERINC3* and *SERINC5* (S5A Fig). However, the infectivity of SIV_mac_239_AAA_ collected from these cells did not differ from SIV_mac_239, indicating that Nef_AAA_ retained the ability to overcome this block. Similar results were obtained for NF-κB stimulation, which is thought to facilitate virus replication by increasing transcription from the LTR promoter [41]. In contrast to Nef_G2A_, Nef_AAA_ retained the ability to stimulate NF-κB signaling to a similar extent as wild-type Nef (S5B Fig). Thus, in addition to CD3-, CD4- and MHC class I-downmodulation, these functions of Nef are not affected by the substitutions in SIV_mac_239_AAA_.

### Sensitivity to tetherin impairs SIV replication during acute infection

**Fig 4:**
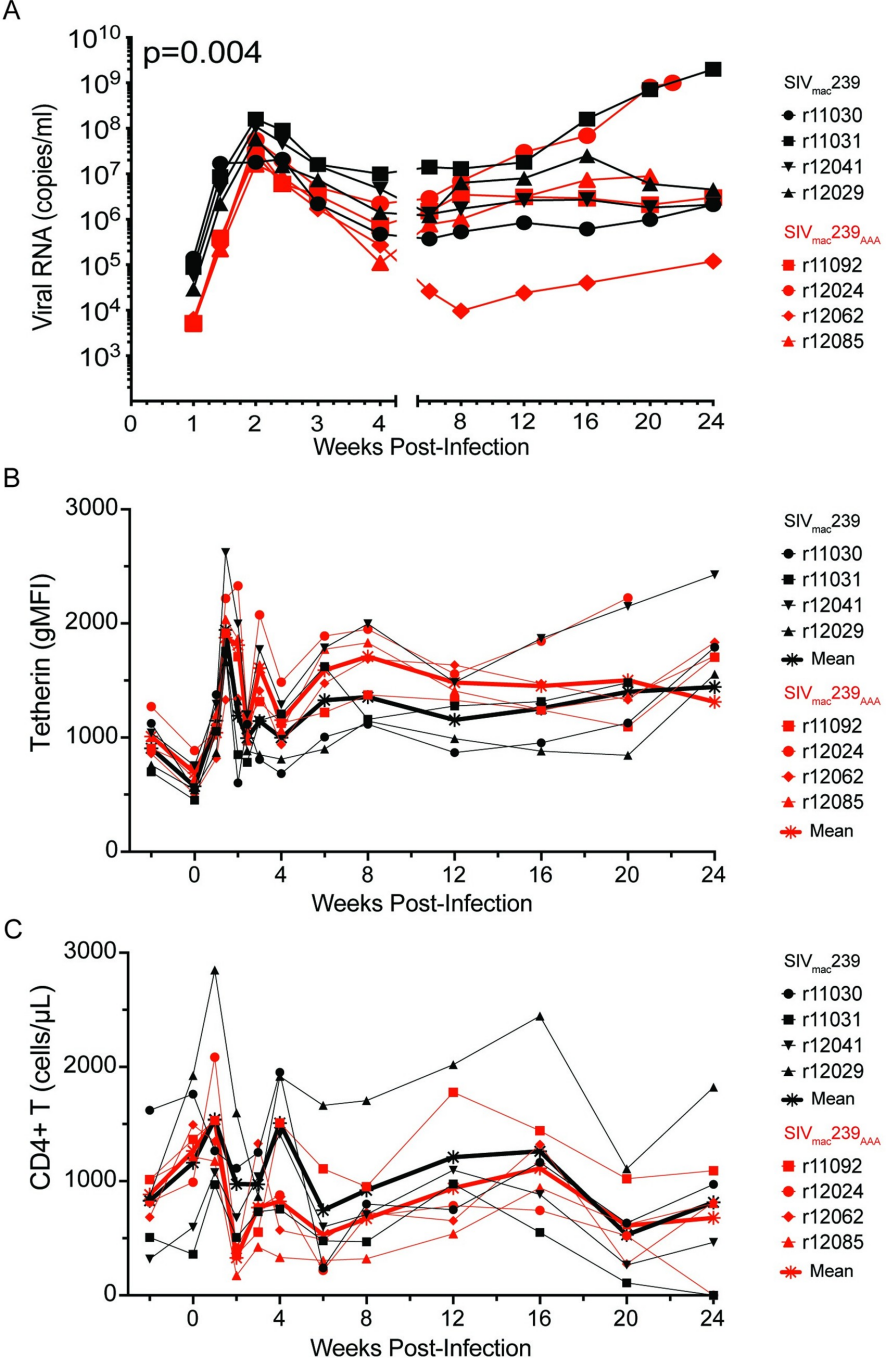
SIV loads in plasma and tetherin upregulation on CD4^+^ lymphocytes in SIV_mac_239_AAA_- versus SIV_mac_239-infected macaques. (A) Viral loads in plasma are shown during acute (weeks 1–4) and chronic (weeks 5–24) infection following intravenous inoculation of macaques with wild-type SIV_mac_239 (black) or SIV_mac_239_AAA_ (red). Viral RNA loads were measured by qRT-PCR using an assay with a threshold of detection of 30 copies/ml. Viral loads were significantly lower in SIV_mac_239_AAA_-infected animals during acute infection (p = 0.004, linear mixed-effects model). Longitudinal changes in tetherin upregulation on memory CD95^+^CCR7^-^CD4^+^ T cells (B) and memory CD95^+^CD4^+^ T cell counts (C) were monitored by flow cytometry. The mean values for animals infected with SIV_mac_239 (black) and SIV_mac_239_AAA_ (red) are indicated by heavier lines.

To assess the contribution of tetherin antagonism to lentiviral replication *in vivo*, separate groups of four rhesus macaques were infected with SIV_mac_239_AAA_ or wild-type SIV_mac_239, and viral RNA loads in plasma were measured at longitudinal time points post-infection. Since tetherin is polymorphic in the rhesus macaque [42], these animals were also genotyped for tetherin (S1 Table) and the sensitivity of SIV_mac_239_AAA_ to each of the tetherin alleles expressed by animals infected with this virus was verified. The tetherin alleles present in these animals (rBST-2.1, rBST-2.2, rBST-2.6 and rBST-2.14) all inhibited the release of SIV_mac_239_AAA_ to a similar extent as SIV_mac_239Δ*nef* (Fig 2A & S6 Fig). Viral load comparisons revealed significantly lower viremia in SIV_mac_239_AAA_-infected animals than in SIV_mac_239-infected animals during acute infection (*p* = 0.004, mixed-effects model, weeks 1–4 PI) (Fig 4A), which is consistent with the rapid upregulation of tetherin on CD4^+^ T cells coincident with peak IFNα levels on day 10 PI (Fig 4B) [43]. Although tetherin expression on CD4^+^ T cells also remained above baseline levels throughout chronic infection (weeks 5–24 PI) (Fig 4B), differences in viral loads were not detectable (Fig 4A). The absence of a detectable difference in chronic phase viral loads in these outbred animals is not surprising, since the more variable effects of adaptive immunity likely obscured the impact of tetherin on virus replication. Accordingly, the rates of CD4^+^ T cell turnover did not differ between SIV_mac_239- and SIV_mac_239_AAA_-infected animals (Fig 4C).

### Sequence changes in Nef during SIV_mac_239_AAA_ replication in macaques confer resistance to tetherin and SERINC5

**Fig 5:**
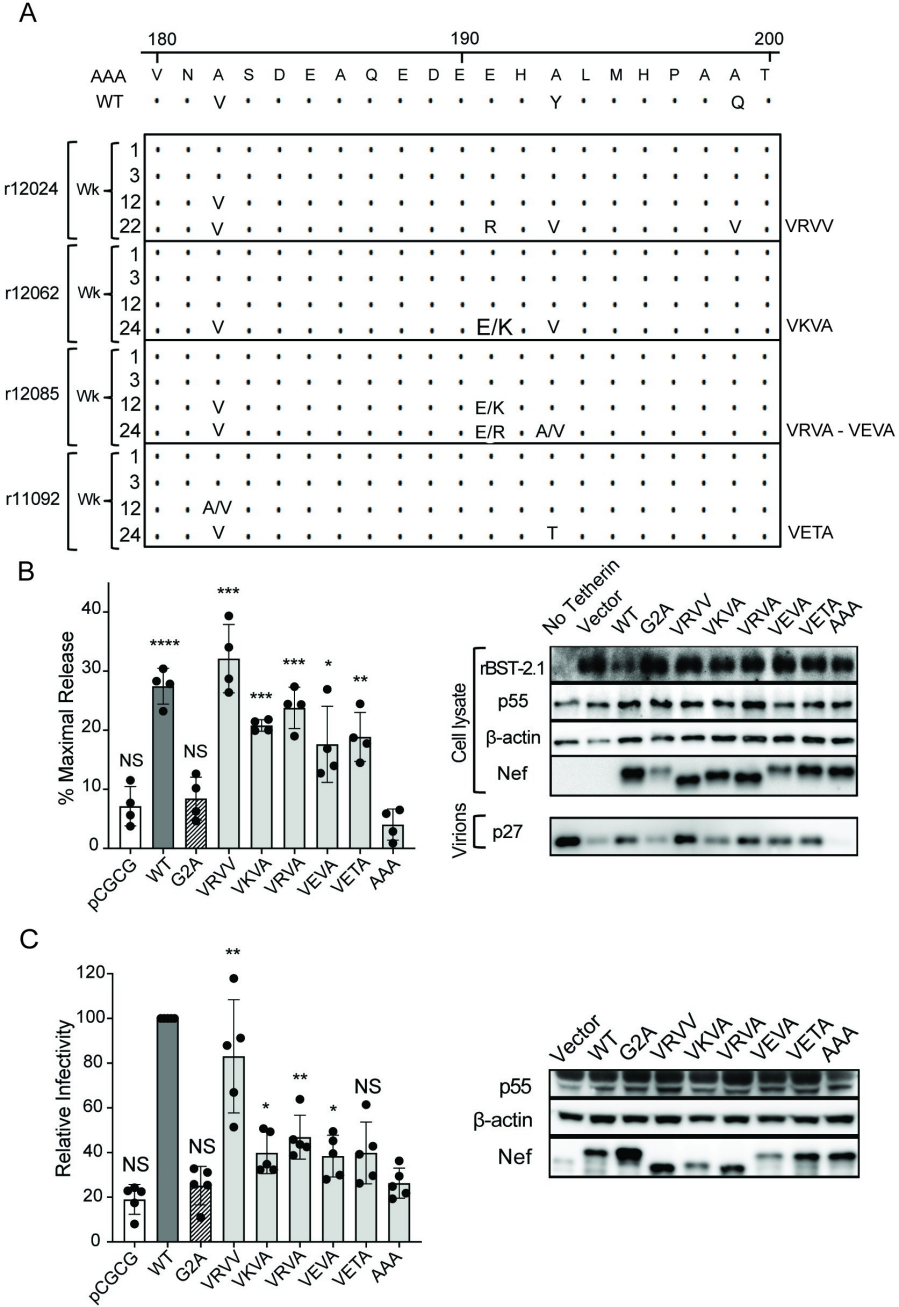
Sequence changes in Nef restore resistance to tetherin and SERINC5. (A) The virus population in plasma of animals infected with SIV_mac_239_AAA_ was sequenced at the indicated time points post-infection. The predicted amino acid sequences in the flexible loop region of Nef are aligned to the corresponding Nef sequences of SIV_mac_239_AAA_ and SIV_mac_239. Positions of identity are indicated by periods and amino acid differences are identified by their single-letter code. (B & C) The Nef variants observed at 22–24 weeks PI in SIV_mac_239_AAA_-infected animals were tested for the ability to counteract tetherin (B) and SERINC5 (C). (B) 293T cells were co-transfected with SIV_mac_239Δ*nef* together with constructs expressing the indicated Nef variants and rhesus macaque tetherin. Percent maximal virus release was calculated from the accumulation of SIV p27 in the culture supernatant of cells transfected with tetherin relative to cells transfected with empty vector. Differences in virus release were corroborated by western blot analysis as described in Fig 1. (C) C8166-SEAP cells were infected with Nef *trans*-complemented virus produced by co-transfection of parental JTAg cells (SERINC3^+^5^+^) with SIV_mac_239Δ*nef* and the indicated Nef variants. Infectivity was measured as described in Fig 3 and Nef expression was confirmed by western blot analysis. (B & C) Error bars indicate standard deviation of the mean for at least four independent experiments and significant differences relative to Nef_AAA_ are indicated by asterisks (*p<0.05, ** *p*<0.01, *** & *p*<0.001, two-tailed unpaired *t*-test with Welch’s correction in case of unequal variance).

Sequence analysis of the virus population in plasma on weeks 1 and 3 PI confirmed that all the SIV_mac_239_AAA_-infected animals retained the V182A, Y193A and Q199A substitutions in Nef during acute infection (Fig 5A). However, by week 12 PI, three of the animals had acquired amino acid changes in Nef, including reversion of the alanine substitution at position 182 to valine (A182V). By weeks 22–24 PI, all four SIV_mac_239_AAA_-infected animals had the A182V reversion and three of the animals also had an alanine-to-valine change at position 193 (A193V), which was associated with an adjacent glutamic acid-to-arginine or lysine change at position 191 (E191R or E191K) (Fig 5A & S7A Fig). Additional changes were observed in two of the animals, including A199V in r12024 and A193T in r11092 (Fig 5A & S7A Fig). Thus, by 22–24 weeks PI the virus population in each of the animals infected with SIV_mac_239_AAA_ accumulated mutations that either restored the wild-type residue or introduced other amino acids at two or more adjacent positions. Env sequences were also compared, since Env has been shown to acquire anti-tetherin activity in macaques infected with SIV_mac_239Δ*nef* [18]. With the exception of a valine-to-methionine substitution at residue 67 (V67M) that also occurs in animals infected with wild-type SIV_mac_239, the amino acid changes in Env differed among the SIV_mac_239_AAA_-infected animals and were scattered throughout the protein (S7B Fig). These changes are therefore unlikely to contribute to tetherin or SERINC5 antagonism.

To determine if the sequence changes in Nef observed in SIV_mac_239_AAA_-infected animals restore resistance to tetherin and/or SERINC5, each of the *nef* alleles identified at weeks 22–24 PI were tested for the ability to *trans-*complement SIV_mac_239Δ*nef* in virus release and infectivity assays. All of the Nef variants rescued virus release significantly better than Nef_AAA_ (Fig 5B). Likewise, all of the Nef variants except VETA significantly increased virus infectivity in comparison to Nef_AAA_ (Fig 5C). However, only one of the variants (VRVV) restored viral infectivity to a level comparable to wild-type Nef (Fig 5C). To confirm that these results accurately reflect the ability to counteract macaque SERINC5, each of the Nef variants were also tested for the ability to rescue SIV_mac_239Δ*nef* infectivity in 293T cell lines that express human or rhesus macaque SERINC5. Consistent with minimal sequence differences between human and macaque SERINC5 and evidence that antagonism of SERINC5 by Nef is not species-specific [39], no differences in activity against human and macaque SERINC5 were detected (S3A Fig). Additional assays confirmed that these Nef variants all retained CD3, CD4, CD28 and MHC-I downmodulation (S8 Fig). Hence, these results indicate that the sequence changes acquired by Nef during SIV_mac_239_AAA_ replication in macaques reflect selective pressure to overcome restriction by tetherin and SERINC5.

## Discussion

Species-specific adaptations of HIV-1 and SIV and studies in mice have consistently indicated that tetherin imposes a strong selective pressure on virus replication [18–20, 44, 45]; however, the contribution of tetherin antagonism to lentiviral replication *in vivo* has not been directly assessed in a primate system. To take advantage of the rhesus macaque as model to address this question, we introduced a combination of mutations into the SIV_mac_239 genome to selectively disrupt the anti-tetherin activity of Nef without affecting other Nef functions. The resulting infectious molecular clone (SIV_mac_239_AAA_) retained the ability to downmodulate CD3, CD4 and MHC class I molecules from the surface of infected cells, but was unable to counteract restriction by tetherin. Infection of macaques with SIV_mac_239_AAA_ resulted in a significant reduction in acute viremia compared to animals infected with wild-type SIV_mac_239 and sequence changes in Nef that restored resistance to tetherin in all animals.

Nef serves as an adaptor for clathrin-mediated endocytosis of a number of transmembrane proteins by stabilizing interactions between the cytoplasmic domains of cellular cargo proteins and subunits of either the AP-1 or AP-2 complexes. MHC class I downmodulation by Nef is dependent on the formation of a ternary complex between Nef, the MHC class I cytoplasmic tail and the μ1 subunit of AP-1 [46]. Nef also binds to the α and σ2 subunits of AP-2 via an interface involving elements of the flexible loop region, including the conserved dileucine motif (ExxxLϕ) [47]. Structural models suggest that this allows Nef to bridge sorting motifs in the cytoplasmic tails of CD3 and CD4 to AP-2 [38]. These features help to explain the difficulty in separating tetherin antagonism from other AP-dependent functions of Nef [15, 48]. Nevertheless, we were able to identify a combination of three amino acid changes in the flexible loop region of Nef that disrupt tetherin antagonism without significant loss of either CD3 or CD4 downmodulation. Additional characterization confirmed that these substitutions also did not disrupt NF-κB stimulation, CD28 downmodulation or the ability to counteract a recently reported block to infectivity in MOLT-3 cells [40], which resembles a block to the replication of HIV-1 and SIV in primary CD4^+^ T cells prior to mitogenic stimulation [49–51].

The substitutions in SIV_mac_239_AAA_ did impair the ability to counteract SERINC5, as indicated by the inability of Nef_AAA_ to restore the infectivity of *nef*-deleted SIV produced in JTAg cells or 293T cells expressing human or rhesus macaque SERINC5. However, in contrast to the nearly complete loss of infectivity for virus produced in these cell lines, the infectivity of SIV_mac_239_AAA_ produced in primary CD4^+^ T cells was only partially impaired. Indeed, SIV_mac_239_AAA_ collected from infected CD4^+^ T cell cultures retained approximately 60% of the infectivity of wild-type SIV_mac_239. Although it is presently unclear what accounts for the greater effect of SERINC5 on virus infectivity in transfected cell lines, it is possible that differences in the timing, relative abundance or subcellular distribution of Nef in transfected cells may have limited the availability of Nef_AAA_ to counteract SERINC5. Nevertheless, the partial impairment of SIV_mac_239_AAA_ infectivity under physiological conditions of Nef_AAA_ expression from the viral genome in SIV_mac_239_AAA_-infected primary CD4^+^ T cells suggests that Nef_AAA_ also retains some activity against SERINC5.

The phenotype of SIV_mac_239_AAA_ is consistent with a recent cryoelectron microscopy structure of SIV_smm_ Nef in complex with the cytoplasmic tail of sooty mangabey tetherin and AP-2 [52]. The V182A, Y193A and Q199A substitutions are all located in a tetherin binding pocket formed by Nef and the β2 subunit of AP-2. V182 directly contacts W17 for the GDIWK sequence that is present in simian tetherin, but not human tetherin. Y193 is adjacent to H192, which also contacts the GDIWK sequence and is essential for binding to tetherin. Furthermore, Q199 is predicted to contact tetherin and an alanine substitution at this position (Q199A) was shown to attenuate tetherin binding [52]. This structure also revealed that although the tetherin binding site is distinct from surfaces of Nef that interact with the cytoplasmic tails of CD3, CD4 and MHC class I molecules, these residues overlap with Nef residues involved in counteracting SERINC5 [52], which helps to explain the difficulty in separating tetherin antagonism from SERINC5 antagonism.

Consistent with the rapid upregulation of tetherin on memory CD4^+^ T cells [43], macaques infected with SIV_mac_239_AAA_ exhibited a significant reduction in acute viremia compared to animals infected with wild-type SIV_mac_239. However, while tetherin expression remained above baseline levels throughout infection, chronic phase viral loads did not differ for SIV_mac_239_AAA_- versus SIV_mac_239-infected animals. The inability to observe a significant difference in viral loads during chronic infection probably reflects the dominant and more variable effects of antibody and T cell responses on SIV replication after the onset of adaptive immunity. Tetherin nevertheless continued to exert selective pressure on virus replication as indicated by the emergence of amino acid changes in Nef that restore anti-tetherin activity after 12–24 weeks of infection. These changes also increased the ability of Nef to counteract SERINC5. However, resistance to SERINC5 was less complete. Whereas all of the Nef variants in circulation by weeks 22–24 PI rescued virus release in the presence of macaque tetherin to a similar extent as wild-type Nef, only one of the variants fully restored the ability to counteract SERINC5.

Although acute viral loads were lower in animals infected with SIV_mac_239_AAA_ than with wild-type SIV_mac_239, they were still considerably higher than in rhesus macaques infected with SIV_mac_239Δ*nef*. In macaques infected with SIV_mac_239Δ*nef* as an attenuated vaccine strain, viral RNA loads in plasma typically peak at about 1x10^5^ copies/ml during acute infection and are often controlled below the limit of detection during chronic infection [53]. Median viral loads in our SIV_mac_239_AAA_-infected animals were 3.2x10^7^ copies/ml at peak (week 2 PI) and ranged from 1x10^6^ to 5x10^6^ copies/ml during chronic infection (weeks 6–24 PI). These values correspond to an expected 2-log difference in peak viremia and a 5- to 6-log difference in chronic phase viral loads between SIV_mac_239_AAA_- and SIV_mac_239Δ*nef*-infected animals, which suggests that other activities of Nef besides tetherin and SERINC5 antagonism have a significant impact on SIV replication.

In summary, we engineered an infectious molecular clone of SIV with mutations in *nef* that render the virus sensitive to restriction by tetherin and SERINC5. Infection of macaques with this virus resulted in a significant reduction in acute viremia compared to animals infected with wild-type SIV and the accumulation of sequence changes in *nef* that restore resistance to tetherin, and to a lesser extent SERINC5. These results demonstrate that overcoming restriction by tetherin and SERINC5 is essential for efficient lentiviral replication in primates.

## Materials and methods

### Ethics statement

Eight rhesus macaques (*Macaca mulatta*) of Indian origin were used in this project. These animals were housed at the Wisconsin National Primate Research Center (WNPRC) in accordance with the standards of the American Association for the Accreditation of Laboratory Animal Care (AAALAC) and the University of Wisconsin Research Animal Resources Center (UWRARC). Animal experiments were approved by the UWRARC (protocol number G005312) and performed in compliance with the principles described in the *Guide for the Care and Use of Laboratory Animals* [54]. Fresh water was always available, commercial monkey chow was provided twice a day and fresh produce was supplied daily. To minimize any pain and distress related to experimental procedures, Ketamine HCL was used to sedate animals prior to blood collection and animals were monitored twice a day by animal care and veterinary staff.

### Plasmid DNA constructs

SIV_mac_239 *nef* and rhesus macaque tetherin (BST-2) alleles were cloned into pCGCG and pcDNA3.1, respectively [12, 18]. Full-length proviral DNA clones for SIV_mac_239 and SIV_mac_239Δ*nef* were obtained as previously described [15, 55, 56]. Nucleotide changes were introduced into SIV *nef* and the SIV_mac_239 genome using the Q5 mutagenesis kit (New England Biolabs). Full-length *SERINC5* cDNA sequences were amplified from RNA extracted from human and rhesus macaque PBMCs by RT-PCR and cloned into the *Not* I and *Bam* HI sites of pQCXIH. Sequences encoding an HA epitope tag were introduced into an extracellular loop of SERINC5 between residues 290 and 291 as previously described [57]. Plasmid DNA constructs for the expression of firefly luciferase from a promoter with three NF-κB binding sites and Gaussia luciferase were provided by Dr. Daniel Sauter (Ulm University Medical Center, Ulm, Germany) [41]. Plasmid DNA constructs were confirmed by Sanger sequencing.

### Cell lines

Jurkat T antigen (JTAg) cells and derivatives with knockout mutations in *SERINC3* (SERINC3^-^5^+^), *SERINC5* (SERINC3^+^5^-^) or both (SERINC3^-^5^-^) [30], and MOLT-3 cells with knockout mutations in *SERINC5* or both *SERINC3* and *SERINC5* [30] were provided by Dr. Heinrich Gottlinger (University of Massachusetts Medical School, Worcester, MA). MOLT-3 cell lines expressing CCR5 were established by retroviral transduction with pCXpur-synCCR5 (also provided by Dr. Gottlinger) followed by selection for puromycin resistant cells. These cell lines were maintained in RPMI 1640 supplemented with 10% fetal bovine serum (FBS), 100 U/ml penicillin, 100 μg/ml streptomycin, 0.25 μg/ml amphotericin B and 2mM L-glutamine (R10 medium). 293T cells were obtained from ATCC and maintained in DMEM medium supplemented with 10% FBS, 100 U/ml penicillin, 100 μg/ml streptomycin, 0.25 μg/ml amphotericin B and 2mM L-glutamine (D10 medium). Stable 293T cell lines expressing HA-tagged human or rhesus macaque SERINC5 were established by retroviral transduction with pQCXIH vectors expressing human or macaque SERINC5 and maintained in medium with 100 μg/ml hygromycin. TZM-bl cells were obtained through the NIH AIDS Reagent Program, Division of AIDS, NIAID, NIH: TZM-bl cells from Dr. John C. Kappes, and Dr. Xiaoyun Wu, and were cultivated in D10 medium [58–62]. C8166-SEAP cells were maintained in R10 medium supplemented with 50 μg/ml G418 [63]. Rhesus macaque peripheral blood mononuclear cells (PBMCs) were isolated from whole blood by separation on Ficoll-Paque gradients. PBMCs were activated in R10 medium containing concanavalin A (5 μg/ml) for three days, washed and resuspended in R10 medium supplemented with recombinant IL-2 (20 U/ml).

### Tetherin genotyping

Total RNA was isolated from rhesus macaque PBMCs using the RNeasy Mini Kit (Qiagen). Tetherin cDNA was synthesized from RNA and amplified using the SuperScript III One-Step RT-PCR System (Invitrogen) with primers 5’-CCTTCAGCTAGAGGGGAGATCTGGATG-3’ (sense) and 5’-GACCAGCTTCCTGGGATCTCACAGC-3’ (antisense) according to the manufacturer’s protocol. Tetherin cDNA amplicons were T/A cloned into pGEM-T easy vector (Promega) and confirmed by Sanger sequencing. At least 10 cDNA clones were sequenced to determine the genotype of each animal.

### Sequencing

Viral RNA was isolated from plasma and subjected to genome-wide deep sequencing, as previously described [64]. Briefly, viral RNA was reverse transcribed and the viral cDNA was PCR amplified with the Superscript III one-step reverse transcription PCR (RT-PCR) system with using high-fidelity Platinum *Taq* (Invitrogen). Four overlapping amplicons were generated that spanned the entire SIV coding sequence. PCR products were purified by the MinElute gel extraction kit (Qiagen), and quantified using the Quant-IT double-stranded DNA (dsDNA) HS assay kit (Invitrogen). The four amplicons were pooled to a total of 1 ng and libraries were generated and tagged with barcodes using the Nextera XT kit (Illumina). The tagged libraries were quantified with the Quant-IT dsDNA HS assay kit, and the quality of the libraries was analyzed with an Agilent Bioanalyzer. Pooled libraries were then sequenced on an Illumina MiSeq instrument.

### Quantitative RT-PCR analysis of SERINC3 and SERINC5 mRNA transcripts

The Qiagen RNeasy Mini Kit was used to isolate RNA from JTAg cells and from rhesus macaque primary CD4^+^ cells. cDNA was synthesized using SuperScript III First-Strand Synthesis System (Invitrogen) according to the manufacturer’s protocol. Quantitative RT-PCR was performed using an ABL 7500 instrument and KAPA PROBE FAST per the manufacturer’s instructions using the primers specific for rhesus macaque *SERINC3*, *SERINC5 and GAPDH* shown in S2 Table. Relative *SERINC3* and *SERINC5* mRNA copies were determined as previously described [65] and normalized to *GAPDH* mRNA.

### Virus release assay

293T cells were seeded at 5x10^4^ cells per well in 24-well plates in 0.5 ml D10 medium. Cells were co-transfected the following day with a full-length SIV_mac_239Δ*nef* clone (100 ng), pcDNA3.1-rBST-2 (50 ng) and either pCGCG, pCGCG-239-Nef or pCGCG-239-Nef mutants (100 ng). Differences in the amount of pcDNA3.1-rBST-2 were offset by the addition of empty pcDNA3.1 vector. Transfections were performed in duplicate using GenJet In Vitro DNA Transfection Reagent (Ver. II) (SignaGen Laboratories) according to the manufacturer’s instructions. Forty-eight hours post-transfection, the amount of virus released into the cell culture supernatant was measured by SIV p27 antigen-captured ELISA (Advanced Bioscience Laboratories. Inc.). Virus release was calculated at the percentage of maximal p27 release relative to control transfections in the absence of tetherin as previously described [12].

### Infectivity assays

JTAg cells and derivatives with knock-out mutations in *SERINC3* and/or *SERINC5* (1 x10^5^ cells) were co-transfected with SIV_mac_239Δ*nef* (450 ng), and either pCGCG, pCGCG-239-Nef or pCGCG-239-Nef mutants (50 ng), using GenJet In Vitro DNA Transfection Reagent for Jurkat Cells (SignaGen Laboratories). At 72 h post transfection, the supernatant was collected, and SIV p27 capsid was measured by SIV antigen-capture ELISA. C8166 cells containing a SIV Tat-inducible secreted alkaline phosphatase (SEAP) reporter gene [63] were infected in triplicate wells of 96-well plates with Nef-*trans*-complemented virus (0.5 ng p27 per 1 x10^4^ cells) and the SEAP activity in the cell culture supernatant was measured 72 hours later [63]. The infectivity of SIV_mac_239Δ*nef* produced in the absence of Nef (pCGCG vector) or in the presence of the indicated SIV Nef mutants is expressed as a percentage of infectivity for SIV_mac_239Δ*nef* produced in the presence of wild-type Nef (pCGCG-239-Nef).

MOLT-3 cells expressing CCR5 (MOLT-3-CCR5) with and without knock-out mutations in *SERINC5* (MOLT-3 S5KO-CCR5) or *SERINC3* and *SERINC5* (MOLT-3 DKO-CCR5) were infected with SIV_mac_239, SIV_mac_239Δ*nef* and SIV_mac_239_AAA_ (50 ng p27 per 1x10^6^ cells). The cells were washed after 24 hours to remove the SIV inoculum and supernatant containing progeny virus was collected on day six post-infection. TZM-bl cells were infected in triplicate wells with virus produced in MOLT-3 cells at 0.5 ng SIV p27 per 1x10^4^ cells, and luciferase activity was measured three days post-infection using BriteLite Plus luciferase substrate (Perkin-Elmer) according to the manufacturer’s instructions.

### NF-κB stimulation

293T cells (2 x10^4^ cells) were co-transfected in triplicate wells of 96-well plates with Nef expression constructs (25 ng), a firefly luciferase reporter under the control of promoter with three NF-κB binding sites (50 ng) and a construct that constitutively expresses Gaussia luciferase (50 ng). The next day, the cells were stimulated with TNFα (20 ng/ml) in fresh D10 medium. The following day (48 hours post-transfection), firefly luciferase activity was measured in cell lysates using BriteLite Plus luciferase substrate and Gaussia luciferase was measured in the cell culture supernatant using the Pierce Gaussia Luciferase Flash assay (Thermo Scientific). To control for variation in the efficiency of transfection, firefly luciferase activity was normalized to Gaussia luciferase activity in each well.

### Immunoblotting

Cell culture supernatant was collected and cell lysates were prepared in RIPA buffer containing 1x Halt protease inhibitor. Virons were recovered from the cell culture supernatant by centrifugation at 13,000 rpm for 3 hours at 4°C. Cell lysates and virions were resuspended in Laemmli buffer containing 5% β-mercaptoethanol and boiled for 10 minutes prior to protein separation by electrophoresis on 12% SDS polyacrylamide gels and transfer to polyvinylidine fluoride (PVDF) membranes. Membranes were blocked in 5% non-fat dry-milk in 1x PBS 0.05% Tween-20 for an hour at room temperature, then probed over night at 4°C with primary antibody diluted 1:1000 in 0.5% non-fat dry milk PBS -0.05% Tween-20. The SIV p55 and p27 Gag proteins were detected using mouse monoclonal antibody 183-H12-5C (NIH AIDS Reagent Program). SIV Nef was detected with the mouse monoclonal antibody 17.2 (NIH AIDS Reagent Program). β-actin was detected with the mouse monoclonal antibody C4 (Merck Millipore). Rhesus macaque tetherin (BST-2) was detected using a mouse polyclonal antibody (Abcam). Hsp90 was detected with the mouse monoclonal antibody sc-13119 HRP (Santa Cruz Biotechnology). Membranes were washed three times with PBS -0.05% Tween-20 and probed with goat anti-mouse IgG (H+L) HRP-conjugated secondary antibody (BioRad) for one hour at room temperature. Blots were washed three times with PBS -0.05% Tween-20, treated with Clarity Western ECL substrate (BioRad) for 10 seconds and imaged using Fujifilm Image Reader LAS 3000 (Fujifilm Photo Film Co.).

### Preparation of SIV challenge stocks and infection of rhesus macaques

Challenge stocks were prepared by transfecting 293T cells with full-length infectious molecular clones of SIV_mac_239 and SIV_mac_239_AAA_. Cell culture supernatant was collected on day 2 post-transfection and virus yields were determined by SIV p27 antigen-capture ELISA. The infectivity titers of the virus stocks were determined by limiting dilution on CEMx174 cells.

Separate groups of four rhesus macaques were inoculated intravenously with SIV_mac_239 and SIV_mac_239_AAA_. Thirty minutes prior to challenge, cryopreserved vials of each virus stock were thawed, diluted to 500 TCID_50_ (0.4 ng p27) per ml in serum-free RPMI and loaded into one ml syringes. Animals were sedated with ketamine and dexmedetomidine (5 mg/kg and 0.015 mg/kg, IM), and one ml of the virus dilution was administered to each animal through a 22-24-gauge catheter placed aseptically in the saphenous vein. Sedation was reversed with atipamezole (0.15 mg/kg, IM).

### Plasma viral RNA load measurements

Plasma was separated from blood drawn in tubes with EDTA as an anticoagulant and cryopreserved at -80°C. Viral particles were pelleted from 0.5 to 1.0 ml of plasma by centrifugation for one hour at 20,000 x g. Viral RNA was extracted from virion pellets, reverse- transcribed into cDNA and measured using a real-time PCR assay based on quantitative amplification of a conserved sequence in the SIV *gag* gene as described previously [66].

### Flow cytometry

*Tetherin expression and memory CD4*^*+*^ *T cell counts*. PBMCs (2 x10^6^ cells) were stained for 20 minutes at room temperature with anti-CD8 Amcyan (clone SK1), anti-CD16 PB (clone 3G8), anti-CCR7 FITC (clone 150503), anti-CD28 PE (CD28.2), anti-CD3 PE-CF594 (clone Sp34-2), anti-CD95 PE-Cy5 (DX2), anti-CD4 PE-Cy7 (OKT4), anti-Tetherin APC (clone RS28E), anti-CD20 Alexa700 (clone 2H7) and NearIR live/dead cell stain (Invitrogen). PBMCs were also stained with an isotype control for anti-Tetherin APC. Samples were fixed in 2% paraformaldehyde PBS and analyzed using a BD LSRII SORP instrument. The geometric mean florescence intensity of tetherin staining on CD95^+^CCR7^-^CD4^+^ T cells was measured relative to staining with the isotype control antibody. At each time point, the number of lymphocytes in circulation was also determined by complete blood count (CBC) analysis. Memory CD4^+^ T cell counts per μl of blood were calculated by multiplying the number of lymphocytes per μl blood by the percentage of CD95^+^CD4^+^CD3^+^ lymphocytes at each time point. Flow cytometry data was analyzed using FlowJo 9.9 software (TreeStar Inc.).

*Surface protein downmodulation*. Jurkat and TZM-bl cells (5x10^5^ cells) were transfected with pCGCG constructs that co-express SIV Nef and enhanced green fluorescence protein (GFP) from a downstream internal ribosome entry site (100 ng) using GenJet In Vitro DNA Transfection Reagent for Jurkat Cells and HeLa Cells, respectively (SignaGen Laboratories). Three days post transfection, TZM-bl cells were stained with a PE-Cy7-conjugated monoclonal antibody to CD4 (OKT4, BD Pharmingen) and Jurkat cells were stained with a PE-conjugated monoclonal antibody to MHC-I (HLA-ABC, Dako). After gating on GFP^+^ cells, the geometric mean fluorescence intensities (gMFI) of CD4 and MHC-I expression were determined by flow cytometry.

For analysis of primary cells, rhesus macaque PBMCs were activated for three days with concanavalin A (5 μg/ml) in R10 medium. CD4^+^ lymphocytes were isolated by positive selection using immunomagnetic beads and unconjugated anti-CD4 antibody (OKT4). One million CD4^+^ T cells were infected with SIV_mac_239, SIV_mac_239Δ*nef* or SIV_mac_239_AAA_ and maintained in R10 medium with IL-2 (20 U/ml) for five days. On day 6 post-infection, the cells were stained with anti-tetherin APC (clone RS28E), anti-CD3 PE-CF594 (clone Sp34-2), anti-CD4 PE-Cy7 (OKT4), anti-MHC-I PE, anti-SIV Gag FITC (55-2F12) and NearIR live/dead cell stain (Invitrogen). After gating on the SIV-infected (Gag^+^CD4^lo^) population, the geometric mean fluorescence intensities (gMFI) of BST-2, CD3, CD4 and MHC-I staining were determined. Flow cytometry data was collected using a BD LSRII SORP instrument and analyzed using FlowJo 9.9 software (TreeStar Inc.)

### Statistical analysis

A linear mixed-effect model was used to compare differences in viral loads between rhesus macaques infected with SIV_mac_239_AAA_ and SIV_mac_239. An individual macaque was considered as a random-effect to account for correlation of measurements within subjects. The presence or absence of anti-tetherin activity and the time points after infection were considered as fixed-effects in the model. The three phases of infection were pre-infection (week 0), acute infection (weeks 1–4) and chronic infection (weeks 5–24). All *in vitro* data were analyzed using a two-tailed unpaired *t*-test with Welch’s correction in case of unequal variance.

## Supporting information

S1 Fig CD4 and MHC-I downmodulation by SIV Nef mutants. TZM-bl (A) and Jurkat cells (B) were transfected with pCGCG constructs that co-express SIV Nef and enhanced green fluorescent protein (GFP) from a downstream internal ribosomal entry site. Three days post-transfection, TZM-bl cells were stained with a PE-Cy7-conjugated antibody to CD4 (OKT4, BD Pharmingen) and Jurkat cells were stained with an PE-conjugated antibody to MHC-I (HLA-ABC, Dako). Differences in the geometric mean fluorescence intensities (gMFI) of CD4 (A) and MHC class I (B) staining were determined after gating on the transfected (GFP^+^) cells.
(TIF)

S2 Fig Surface expression of CD3, CD4, MHC I and tetherin on primary rhesus macaque CD4^+^ T cells infected with SIV_mac_239_AAA_, SIV_mac_239 and SIV_mac_239Δ*nef*. Activated CD4^+^ lymphocytes from six different animals were infected with SIV_mac_239_AAA_, SIV_mac_239 and SIV_mac_239Δ*nef*. On day six post-infection, the cells were stained for surface expression of CD4, CD3, MHC I and tetherin and for intracellular expression of the SIV Gag protein. Histogram plots show differences in the fluorescence intensity of CD4, CD3, MHC I and tetherin staining on SIV-infected (Gag^+^CD4^lo^) cells.
(TIF)

S3 Fig SIV Nef variants exhibit similar activity against human and rhesus macaque SERINC5. (A) Stable 293T cell lines that constitutively express HA-tagged human or rhesus macaque SERINC5 were co-transfected with SIV_mac_239Δ*nef* and pCGCG constructs expressing the indicated Nef variants. Cell culture supernatant was collected 48-hours post-transfection, virus concentrations were measured by SIV p27 antigen-capture ELISA, and TZM-bl cells were infected in triplicate with equivalents doses of each virus (0.5 ng p27 per 1x10^4^ cells). Luciferase activity was measured in the cells on day three post-infection. Relative infectivity is shown as a percentage of the infectivity of SIV_mac_239Δ*nef trans*-complemented with wild-type Nef. Error bars indicate standard deviation of the mean for five independent experiments. (B) Nef expression relative to Hsp-90 was verified by western blot analysis of cell lysates. (C) Surface expression of human versus rhesus macaque SERINC5 was compared by staining stable HA-SERINC5-transduced 293T cell lines and parental 293T cells with a HA-specific monoclonal antibody.
(TIF)

S4 Fig Relative abundance of *SERINC3* and *SERINC5* mRNA in cell lines and primary CD4^+^ T cells. RNA was extracted from JTAg cells, 293T cells and positively selected rhesus macaque CD4^+^ lymphocytes. Quantitative RT-PCR was performed using an ABI 7500 instrument and primers and probes specific for rhesus *SERINC3*, *SERINC5 and GAPDH* (S2 Table). Error bars indicate standard deviation of the mean for *SERINC3* and *SERINC5* mRNA levels relative to *GAPDH* mRNA for three independent experiments.
(TIF)

S5 Fig The substitutions in Nef_AAA_ do not impair the infectivity of virus produced in MOLT-3 cells or the stimulation of NF-κB. (A) MOLT-3 cells expressing CCR5 (MOLT-3-CCR5) with and without knock-out mutations in *SERINC5* (MOLT-3 S5KO-CCR5) or *SERINC3* and *SERINC5* (MOLT-3 DKO-CCR5) were infected with SIV_mac_239, SIV_mac_239Δ*nef* and SIV_mac_239_AAA_. Supernatant was collected on day 6 post-infection, SIV p27 concentrations were measured by antigen-capture ELISA, and TZM-bl cells were infected in triplicate with an equivalent amount of each virus (0.5 ng SIV p27 per 1x10^4^). On day 3 post-infection, luciferase activity in virus-infected TZM-bl cells was measured and normalized to cells infected with wild-type SIV_mac_239. Error bars indicate standard deviation of the mean for four independent experiments. (B) 293T cells were co-transfected with Nef expression constructs (Nef, Nef_G2A_ or Nef_AAA_), a firefly luciferase reporter construct under the control of promoter with three NF-κB binding sites, and a construct that constitutively expresses Gaussia luciferase. The next day, the cells were stimulated with TNFα (20 ng/ml) in fresh medium. The following day, firefly and Gaussia lucifase activity were measured in cell lysates and cell culture supernatant, respectively. Firefly luciferase was normalized to Gaussia luciferase to control for differences in the efficiency of transfection. Error bars indicate standard deviation of the mean for at least three independent experiments and significant differences relative to Nef_WT_ are indicated by asterisks (*p<0.05, ** *p*<0.01, *** & *p*<0.001, two-tailed unpaired *t*-test with Welch’s correction in case of unequal variance).
(TIF)

S6 Fig Inhibition of virus release by the tetherin alleles expressed by SIV_mac_239_AAA_-infected macaques. 293T cells were co-transfected with SIV_mac_239, SIV_mac_239Δ*nef* and SIV_mac_239_AAA_ together with increasing amounts of constructs expressing the tetherin alleles rBST-2.2, rBST-2.6 and rBST-2.14. The accumulation of SIV p27 in the cell culture supernatant was measured by antigen-capture ELISA and percent maximal virus release was calculated relative to control transfections in the absence of tetherin. Differences in virus release were corroborated by straining immunoblots of virions and cell lysates with antibodies to tetherin, β-actin and to the SIV Gag p55 and p27 proteins.
(TIF)

S7 Fig Nef and Env sequences in SIV_mac_239_AAA_-infected animals. Viral RNA was extracted from plasma and subjected to full-length sequencing using an Illumina MiSeq instrument as previously described [64]. The predicted amino acid sequences for Nef (A) and Env (B) at weeks 22 (r12024) and 24 (r12062, r12085 & r11092) post-infection are aligned to the wild-type Nef and Env sequences of SIV_mac_239. Positions of amino acid identity are indicated with a period, differences are identified by their single-letter amino acid code, and deletions are indicated with a dash.
(PDF)

S8 Fig Nef variants selected in SIV_mac_239_AAA_-infected animals retain CD3-, CD4- CD28- and MHC class I-downmodulation. JTAg cells transfected with bicistronic pCGCG constructs that express GFP and the indicated Nef variants were stained for surface expression of CD3, CD28 and MHC class I molecules. TZM-bl cells transfected with Nef expression constructs were stained for surface expression of CD4. Relative levels of CD3, CD4, CD28 and MHC I staining were determined by comparing the gMFI of staining on GFP^+^ cells expressing Nef to GFP^+^ cells transfected with the empty pCGCG vector at 48 hours post-transfection. Error bars indicate standard deviation of the mean for three independent experiments and significant differences relative to Nef_AAA_ are indicated by asterisks (*p<0.05, ** *p*<0.01 & *** *p*<0.001, two-tailed unpaired *t*-test with Welch’s correction in case of unequal variance).
(TIF)

S1 Table Rhesus macaque tetherin genotypes. The *tetherin* alelles identified in each of the rhesus macaques included in this study (middle) are listed next to their corresponding animal identification numbers (left) and the virus (SIV_mac_239 or SIV_mac_239_AAA_) each animal was infected with (right). The allele designations (*rBST-2*.*x*) correspond to previously reported alleles of rhesus macaque *tetherin* [42].
(DOCX)

S2 Table Quantitative RT-PCR primers and probes for rhesus macaque *SERINC3* and *SERINC5* transcripts. Primers and probes used for measuring the relative abundance of *SERINC3* and *SERINC5* mRNA in human cells lines and primary rhesus macaque CD4^+^ T cells by qRT-PCR.
(DOCX)
