# Supplementary figures and images for: Loss of tetherin antagonism by Nef impairs SIV replication during acute infection of rhesus macaques
Source: PLoS Pathog. 2020 Apr 17;16(4):e1008487. doi: 10.1371/journal.ppat.1008487 (PMC7190186; doi:10.1371/journal.ppat.1008487)

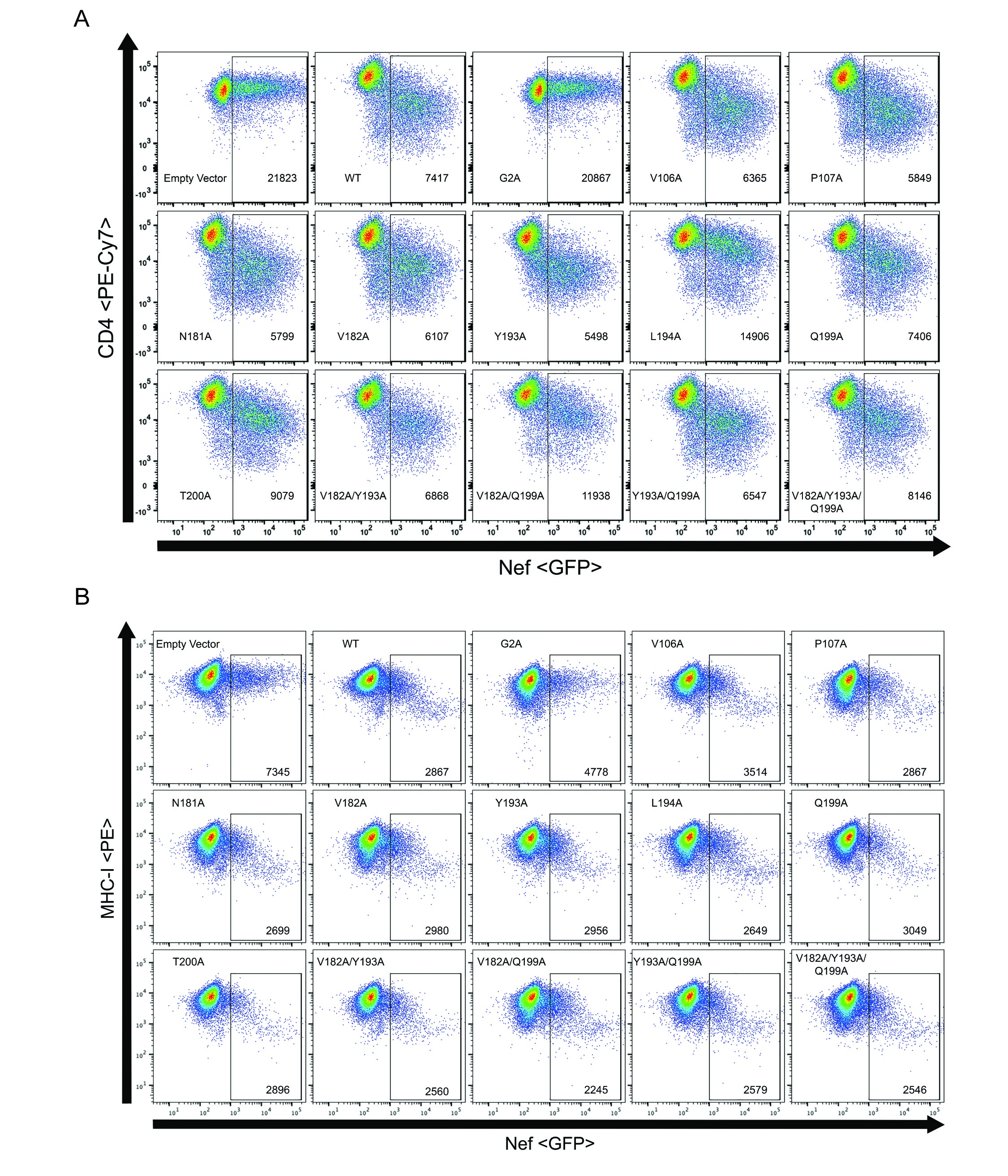

Supplement: S1 Fig — TZM-bl (A) and Jurkat cells (B) were transfected with pCGCG constructs that co-express SIV Nef and enhanced green fluorescent protein (GFP) from a downstream internal ribosomal entry site. Three days post-transfection, TZM-bl cells were stained with a PE-Cy7-conjugated antibody to CD4 (OKT4, BD Pharmingen) and Jurkat cells were stained with an PE-conjugated antibody to MHC-I (HLA-ABC, Dako). Differences in the geometric mean fluorescence intensities (gMFI) of CD4 (A) and MHC class I (B) staining were determined after gating on the transfected (GFP+) cells. (TIF) [file ppat.1008487.s001.tif]

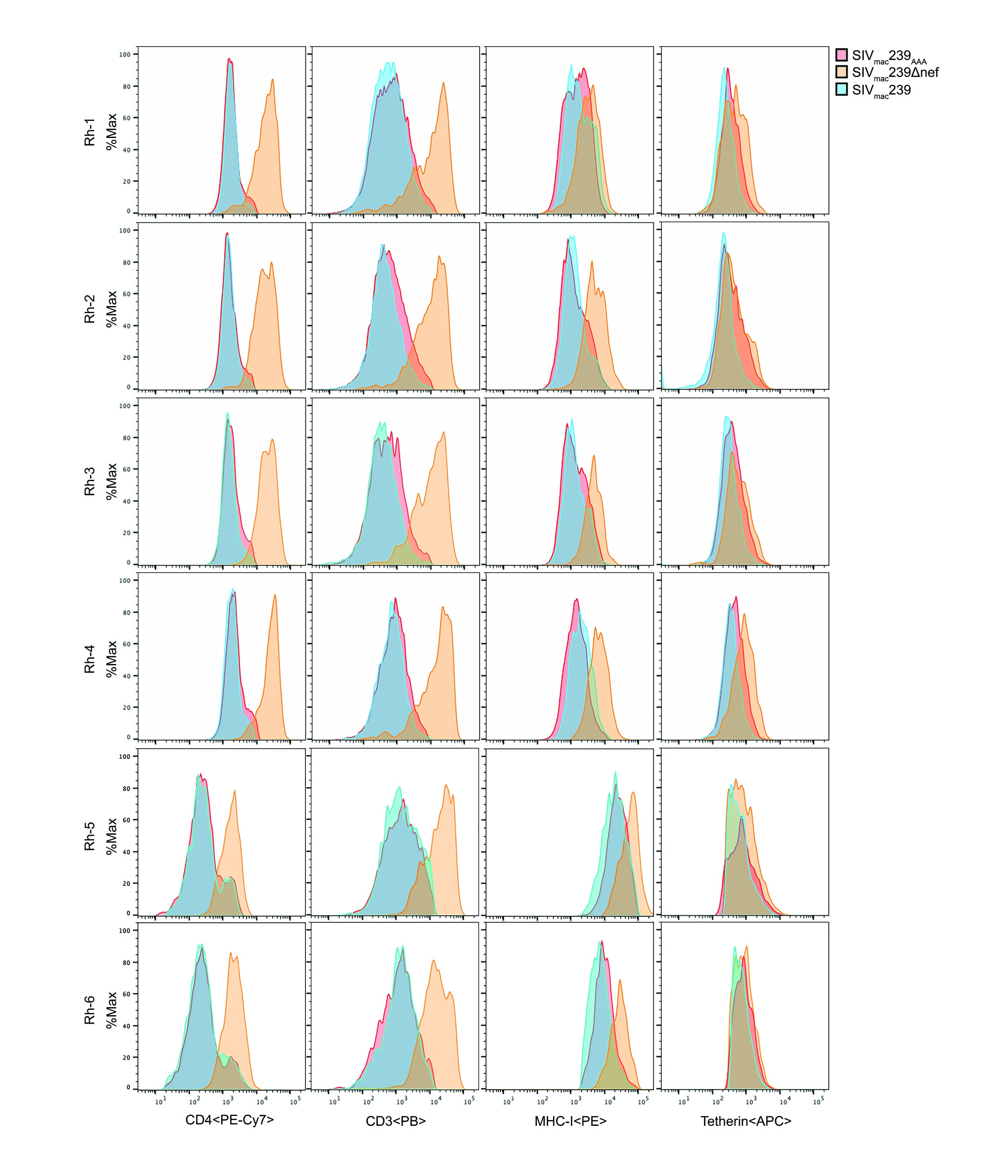

Supplement: S2 Fig — Activated CD4+ lymphocytes from six different animals were infected with SIVmac239AAA, SIVmac239 and SIVmac239Δnef. On day six post-infection, the cells were stained for surface expression of CD4, CD3, MHC I and tetherin and for intracellular expression of the SIV Gag protein. Histogram plots show differences in the fluorescence intensity of CD4, CD3, MHC I and tetherin staining on SIV-infected (Gag+CD4lo) cells. (TIF) [file ppat.1008487.s002.tif]

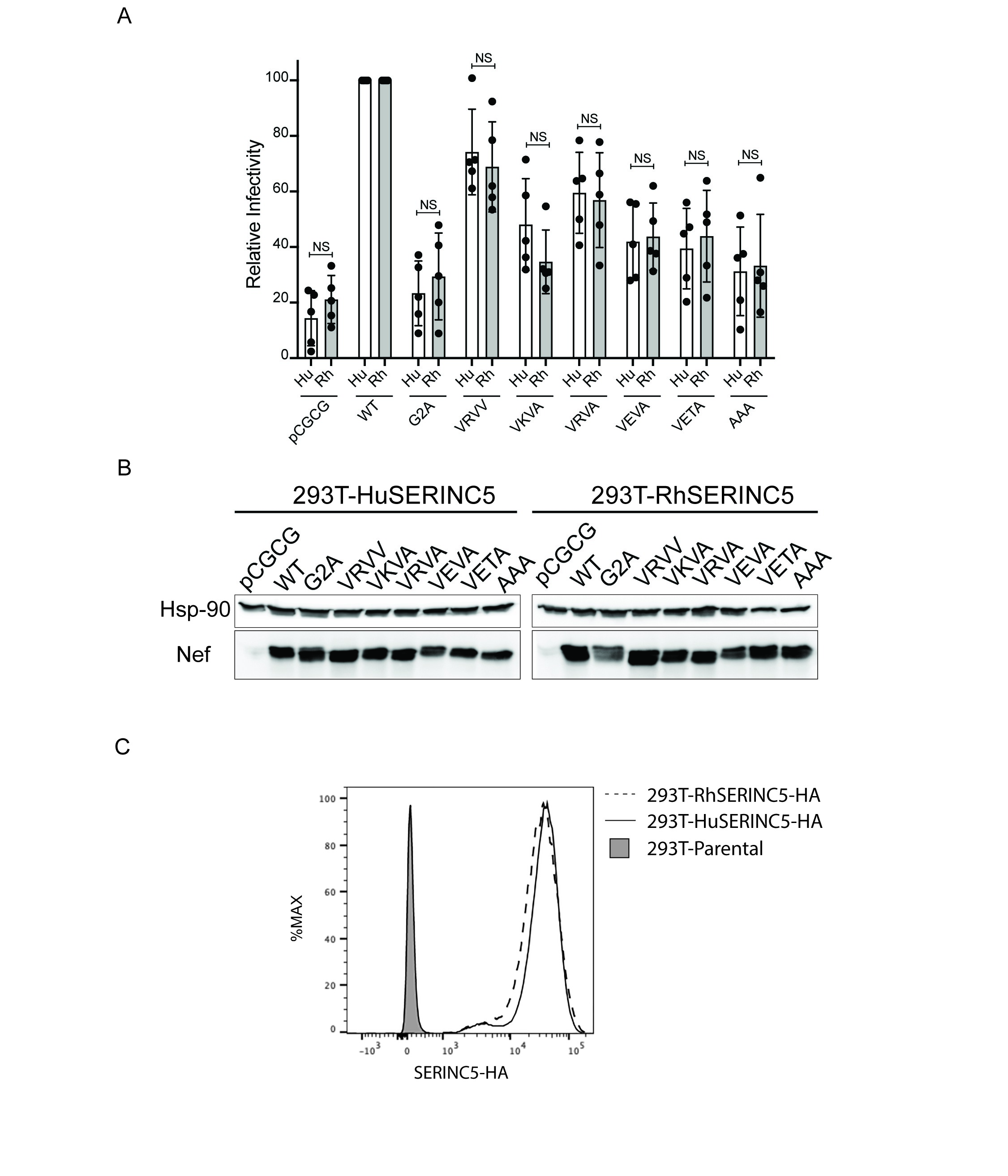

Supplement: S3 Fig — (A) Stable 293T cell lines that constitutively express HA-tagged human or rhesus macaque SERINC5 were co-transfected with SIVmac239Δnef and pCGCG constructs expressing the indicated Nef variants. Cell culture supernatant was collected 48-hours post-transfection, virus concentrations were measured by SIV p27 antigen-capture ELISA, and TZM-bl cells were infected in triplicate with equivalents doses of each virus (0.5 ng p27 per 1x10^4^ cells). Luciferase activity was measured in the cells on day three post-infection. Relative infectivity is shown as a percentage of the infectivity of SIVmac239Δnef trans-complemented with wild-type Nef. Error bars indicate standard deviation of the mean for five independent experiments. (B) Nef expression relative to Hsp-90 was verified by western blot analysis of cell lysates. (C) Surface expression of human versus rhesus macaque SERINC5 was compared by staining stable HA-SERINC5-transduced 293T cell lines and parental 293T cells with a HA-specific monoclonal antibody. (TIF) [file ppat.1008487.s003.tif]

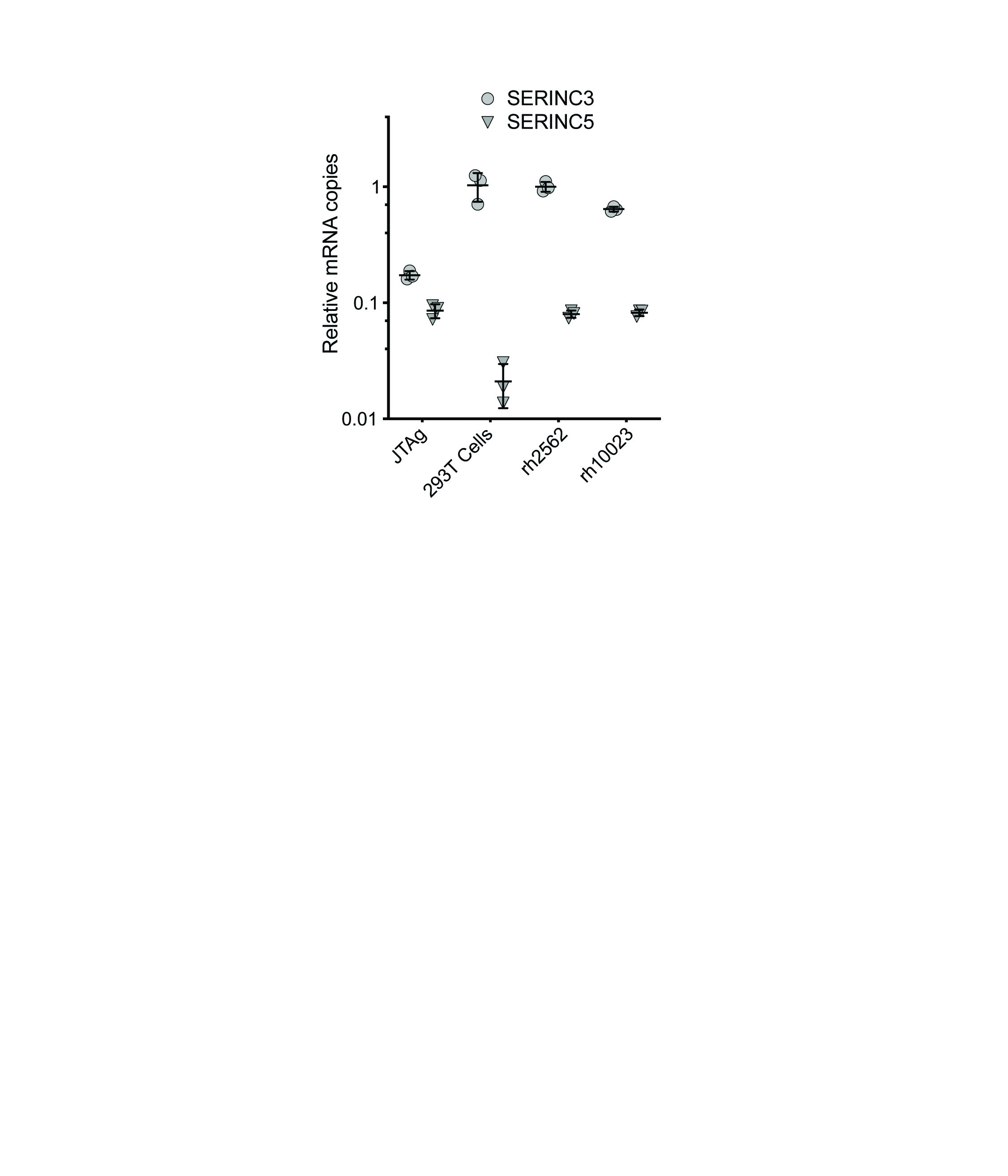

Supplement: S4 Fig — RNA was extracted from JTAg cells, 293T cells and positively selected rhesus macaque CD4+ lymphocytes. Quantitative RT-PCR was performed using an ABI 7500 instrument and primers and probes specific for rhesus SERINC3, SERINC5 and GAPDH (S2 Table). Error bars indicate standard deviation of the mean for SERINC3 and SERINC5 mRNA levels relative to GAPDH mRNA for three independent experiments. (TIF) [file ppat.1008487.s004.tif]

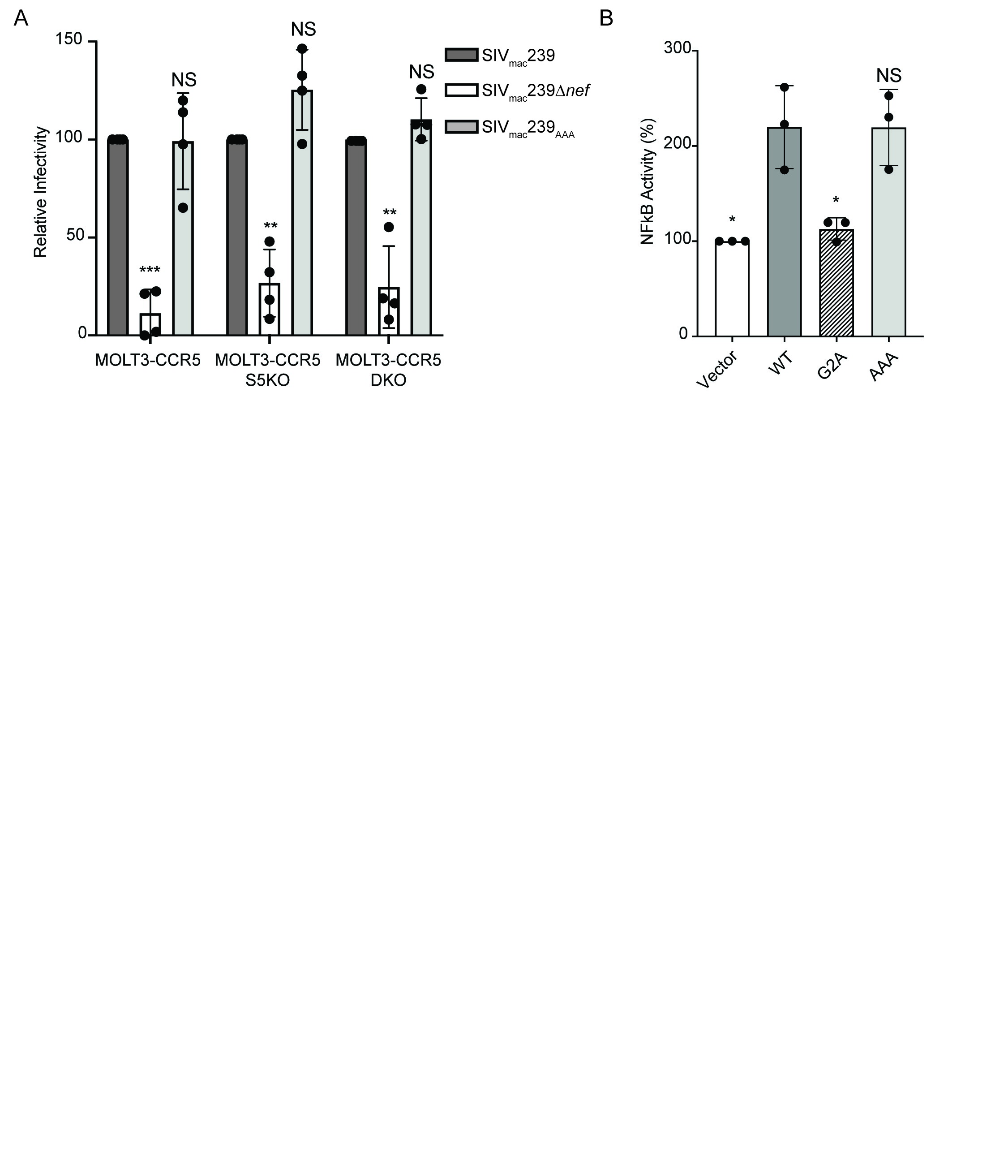

Supplement: S5 Fig — (A) MOLT-3 cells expressing CCR5 (MOLT-3-CCR5) with and without knock-out mutations in SERINC5 (MOLT-3 S5KO-CCR5) or SERINC3 and SERINC5 (MOLT-3 DKO-CCR5) were infected with SIVmac239, SIVmac239Δnef and SIVmac239AAA. Supernatant was collected on day 6 post-infection, SIV p27 concentrations were measured by antigen-capture ELISA, and TZM-bl cells were infected in triplicate with an equivalent amount of each virus (0.5 ng SIV p27 per 1x10^4^). On day 3 post-infection, luciferase activity in virus-infected TZM-bl cells was measured and normalized to cells infected with wild-type SIVmac239. Error bars indicate standard deviation of the mean for four independent experiments. (B) 293T cells were co-transfected with Nef expression constructs (Nef, NefG2A or NefAAA), a firefly luciferase reporter construct under the control of promoter with three NF-κB binding sites, and a construct that constitutively expresses Gaussia luciferase. The next day, the cells were stimulated with TNFα (20 ng/ml) in fresh medium. The following day, firefly and Gaussia lucifase activity were measured in cell lysates and cell culture supernatant, respectively. Firefly luciferase was normalized to Gaussia luciferase to control for differences in the efficiency of transfection. Error bars indicate standard deviation of the mean for at least three independent experiments and significant differences relative to NefWT are indicated by asterisks (*p<0.05, ** p<0.01, *** & p<0.001, two-tailed unpaired t-test with Welch’s correction in case of unequal variance). (TIF) [file ppat.1008487.s005.tif]

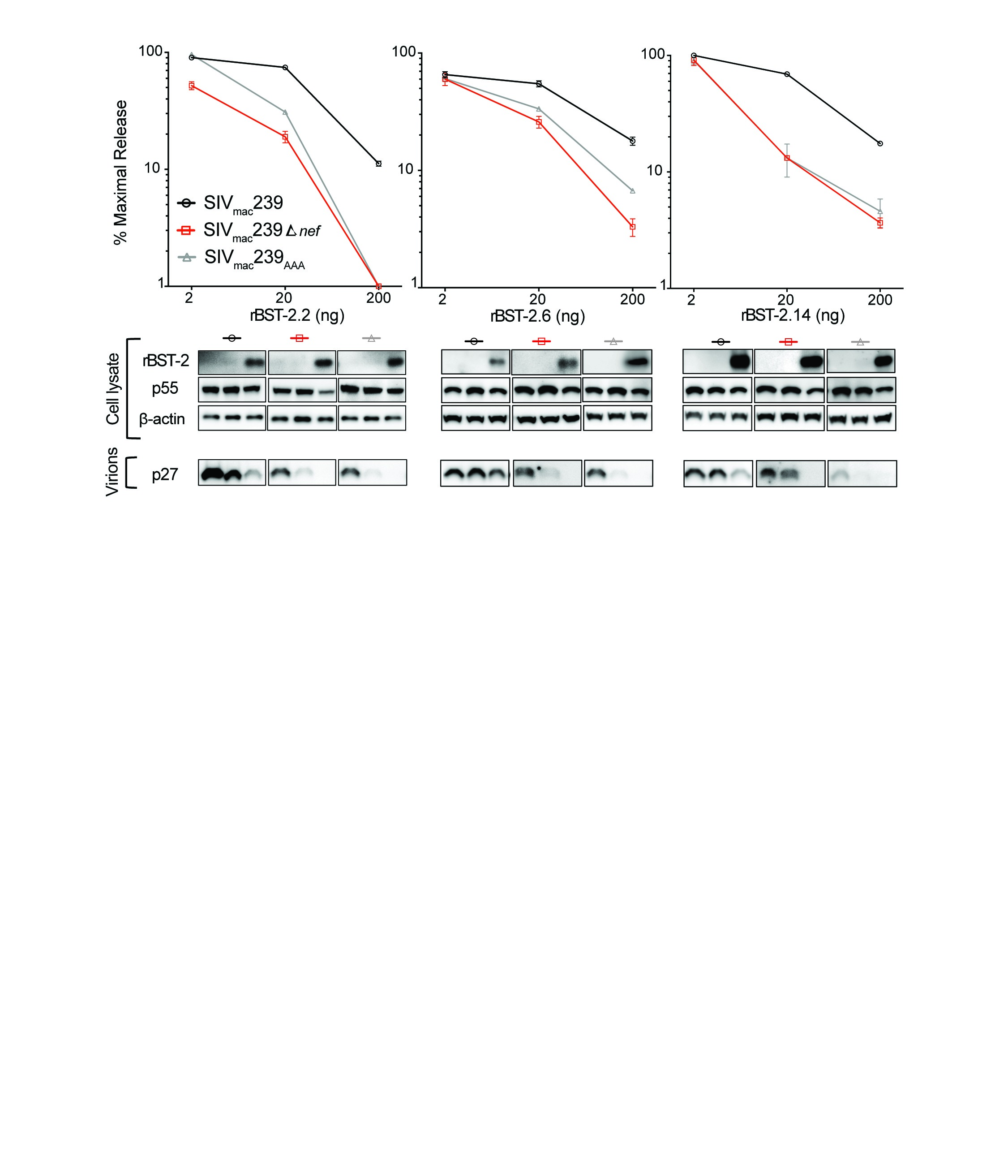

Supplement: S6 Fig — 293T cells were co-transfected with SIVmac239, SIVmac239Δnef and SIVmac239AAA together with increasing amounts of constructs expressing the tetherin alleles rBST-2.2, rBST-2.6 and rBST-2.14. The accumulation of SIV p27 in the cell culture supernatant was measured by antigen-capture ELISA and percent maximal virus release was calculated relative to control transfections in the absence of tetherin. Differences in virus release were corroborated by straining immunoblots of virions and cell lysates with antibodies to tetherin, β-actin and to the SIV Gag p55 and p27 proteins. (TIF) [file ppat.1008487.s006.tif]

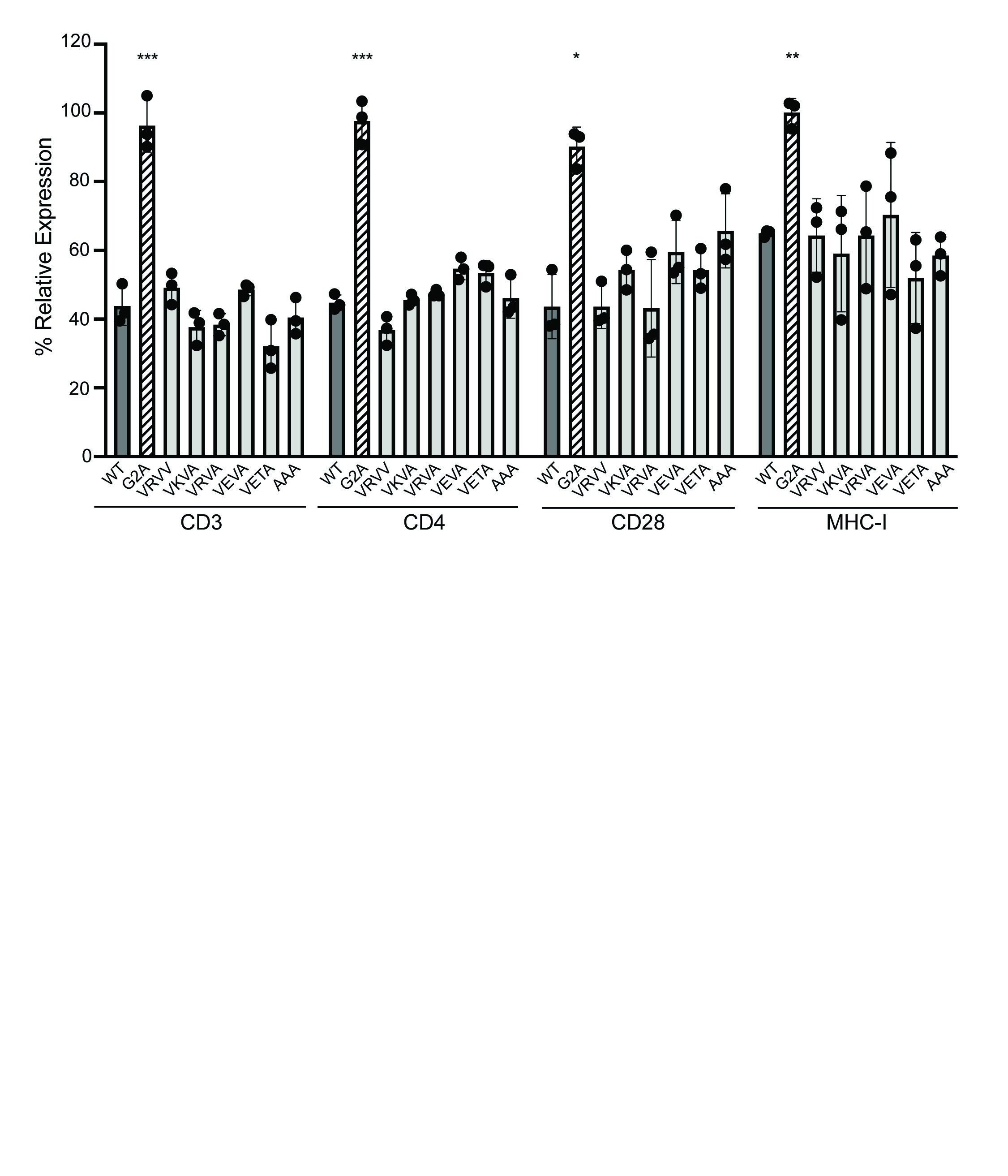

Supplement: S8 Fig — JTAg cells transfected with bicistronic pCGCG constructs that express GFP and the indicated Nef variants were stained for surface expression of CD3, CD28 and MHC class I molecules. TZM-bl cells transfected with Nef expression constructs were stained for surface expression of CD4. Relative levels of CD3, CD4, CD28 and MHC I staining were determined by comparing the gMFI of staining on GFP+ cells expressing Nef to GFP+ cells transfected with the empty pCGCG vector at 48 hours post-transfection. Error bars indicate standard deviation of the mean for three independent experiments and significant differences relative to NefAAA are indicated by asterisks (*p<0.05, ** p<0.01 & *** p<0.001, two-tailed unpaired t-test with Welch’s correction in case of unequal variance). (TIF) [file ppat.1008487.s008.tif]
