# Supplementary material for: Loss of tetherin antagonism by Nef impairs SIV replication during acute infection of rhesus macaques
Source: PLoS Pathog. 2020 Apr 17;16(4):e1008487. doi: 10.1371/journal.ppat.1008487 (PMC7190186; doi:10.1371/journal.ppat.1008487)
Supplement: S7 Fig — Viral RNA was extracted from plasma and subjected to full-length sequencing using an Illumina MiSeq instrument as previously described [64]. The predicted amino acid sequences for Nef (A) and Env (B) at weeks 22 (r12024) and 24 (r12062, r12085 & r11092) post-infection are aligned to the wild-type Nef and Env sequences of SIVmac239. Positions of amino acid identity are indicated with a period, differences are identified by their single-letter amino acid code, and deletions are indicated with a dash. (PDF) [file ppat.1008487.s007.pdf]

A

```
                        10        20        30        40        50        60
SIVmac239 Nef MGGAISMRRSRPSGDLRQRLLRARGETYGRLLGEVEDGYSQSPGGLDKGLSSLSCEGQKY
r12024        ............................................................
r12062        ............................................................
r12085        ............................................................
r11092        ............................................................

                        70        80        90       100       110       120
SIVmac239 Nef NQGQYMNTPWRNPAEEREKLAYRKQNMDDIDEEDDDLVGVSVRPKVPLRTMSYKLAIDMS
r12024        ............................................................
r12062        ............................................................
r12085        ...........................................S................
r11092        ...........................................S/P................

                       130       140       150       160       170       180
SIVmac239 Nef HFIKEKGGLEGIYYSARRHRILDIYLEKEEGIIPDWQDYTSGPGIRYPKTFGWLWKLVPV
r12024        Q...........................................................
r12062        ..............................................F.............
r12085        ............................................................
r11092        ............................................................

                       190       200       210       220       230       240
SIVmac239 Nef NVSDEAQEDEEHYLMHPAQTSQWDDPWGEVLAWKFDPTLAYTYEAYVRYPEEFGSKSGLS
r12024        ..........R.V.....V.........................................
r12062        ..........E/K.V.....A.........................................
r12085        ..........E/R.V/A.....A.........................................
r11092        ............T.....A.........................................

                       250       260
SIVmac239 Nef EEEVRRRLTARGLLNMADKKETR
r12024        .......................
r12062        .......................
r12085        .......................
r11092        .......................
```

B

```
                      10        20        30        40        50        60
SIVmac239 Env MGCLGNQLLIAILLLSVYGIYCTLYVTVFYGVPAWRNATIPLFCATKNRDTWGTTQCLPD
r12024        ............................................................
r12062        ............................................................
r12085        ............................................................
r11092        ............................................................

                      70        80        90       100       110       120
SIVmac239 Env NGDYSEVALNVTESFDAWNNTVTEQAIEDVWQLFETSIKPCVKLSPLCITMRCNKSETDR
r12024        ......M.....................................................
r12062        ......M.....................................................
r12085        ......M.....................................................
r11092        ......M................................................N....

                     130       140       150       160       170       180
SIVmac239 Env WGLTKSITTTASTTSTTASAKVDMVNETSSCIAQDNCTGLEQEQMISCKFNMTGLKRDKK
r12024        .....................................A......................
r12062        ............................................................
r12085        .................T..........................................
r11092        ............................................................

                     190       200       210       220       230       240
SIVmac239 Env KEYNETWYSADLVCEQGNNTGNESRCYMNHCNTSVIQESCDKHYWDAIRFRYCAPPGYAL
r12024        ............................................................
r12062        ............................................................
r12085        .........................................R..................
r11092        ............................................................

                     250       260       270       280       290       300
SIVmac239 Env LRCNDTNYSGFMPKCSKVVVSSCTRMMETQTSTWFGFNGTRAENRTYIYWHGRDNRTIIS
r12024        ................................S...........................
r12062        ............................................................
r12085        ............................................................
r11092        ............................................................

                     310       320       330       340       350       360
SIVmac239 Env LNKYYNLTMKCRRPGNKTVLPVTIMSGLVFHSQPINDRPKQAWCWFGGKWKDAIKEVKQT
r12024        .....................A......................................
r12062        ............................A...............................
r12085        ............................................................
r11092        ............................................................

                     370       380       390       400       410       420
SIVmac239 Env IVKHPRYTGTNNTDKINLTAPGGGDPEVTFMWTNCRGEFLYCKMNWFLNWVEDRNTANQK
r12024        ........................N...................................
r12062        ............................................................
r12085        ......................................................K...H.
r11092        .....................................................------.

                     430       440       450       460       470       480
SIVmac239 Env PKEQHKRNYVPCHIRQIINTWHKVGKNVYLPPREGDLTCNSTVTSLIANIDWIDGNQTNI
r12024        ............................................................
r12062        ............................................................
r12085        ............................................................
r11092        ............................................................
```

```
                       490       500       510       520       530       540
SIVmac239 Env TMSAEVAELYRLELGDYKLVEITPIGLAPTDVKRYTTGGTSRNKRGVFVLGFLGFLATAG
r12024        ............................................................
r12062        ............................................................
r12085        ..............................N.............................
r11092        ..............................N.............................

                       550       560       570       580       590       600
SIVmac239 Env SAMGAASLTLTAQSRTLLAGIVQQQQQLLDVVKRQQELLRLTVWGTKNLQTRVTAIEKYL
r12024        ............................................................
r12062        ............................................................
r12085        ............................................................
r11092        ............................................................

                       610       620       630       640       650       660
SIVmac239 Env KDQAQLNAWGCAFRQVCHTTVPWPNASLTPKWNNETWQEWERKVDFLEENITALLEEAQI
r12024        ............................................................
r12062        ...................A........................................
r12085        ............................I...............................
r11092        ............................................................

                       670       680       690       700       710       720
SIVmac239 Env QQEKNMYELQKLNSWDVFGNWFDLASWIKYIQYGVYIVVGVILLRIVIYIVQMLAKLRQG
r12024        ..............................V.............................
r12062        ............................................................
r12085        ............................................................
r11092        ............................................................

                       730       740       750       760       770       780
SIVmac239 Env YRPVFSSPPSYFQQTHIQQDPALPTREGKERDGGEGGGNSSWPWQIEYIHFLIRQLIRLL
r12024        ............................................................
r12062        ............................................................
r12085        ............................................................
r11092        ............................................................

                       790       800       810       820       830       840
SIVmac239 Env TWLFSNCRTLLSRVYQILQPILQRLSATLQRIREVLRTELTYLQYGWSYFHEAVQAVWRS
r12024        ............................................................
r12062        ..........................T.................................
r12085        ............................................................
r11092        ............................................................

                       850       860       870
SIVmac239 Env ATETLAGAWGDLWETLRRGGRWILAIPRRIRQGLELTLL
r12024        .......................................
r12062        .......................................
r12085        .......................................
r11092        .......................................
```
