# Supplementary material for: Loss of tetherin antagonism by Nef impairs SIV replication during acute infection of rhesus macaques
Source: PLoS Pathog. 2020 Apr 17;16(4):e1008487. doi: 10.1371/journal.ppat.1008487 (PMC7190186; doi:10.1371/journal.ppat.1008487)
Supplement: S1 Table — The tetherin alelles identified in each of the rhesus macaques included in this study (middle) are listed next to their corresponding animal identification numbers (left) and the virus (SIVmac239 or SIVmac239AAA) each animal was infected with (right). The allele designations (rBST-2.x) correspond to previously reported alleles of rhesus macaque tetherin [42]. (DOCX) [file ppat.1008487.s009.docx]

**S1 Table. Rhesus macaque tetherin genotypes.**

| Animal ID | Tetherin Alleles | Virus |
| --- | --- | --- |
| r12024 | *rBST-2.1*  *rBST-2.6* | SIV_mac_239_AAA_ |
| r12062 | *rBST-2.1*  *rBST-2.1* | SIV_mac_239_AAA_ |
| r12085 | *rBST-2.6*  *rBST-2.14* | SIV_mac_239_AAA_ |
| r11092 | *rBST-2.2*  *rBST-2.2* | SIV_mac_239_AAA_ |
| r11030 | *rBST-2.1*  *rBST-2.7* | SIV_mac_239 |
| r11031 | *rBST-2.1*  *rBST-2.1* | SIV_mac_239 |
| r12029 | *rBST-2.1*  *rBST-2.5* | SIV_mac_239 |
| r12041 | *rBST-2.2*  *rBST-2.11* | SIV_mac_239 |
