# Supplementary material for: Loss of tetherin antagonism by Nef impairs SIV replication during acute infection of rhesus macaques
Source: PLoS Pathog. 2020 Apr 17;16(4):e1008487. doi: 10.1371/journal.ppat.1008487 (PMC7190186; doi:10.1371/journal.ppat.1008487)
Supplement: S2 Table — Primers and probes used for measuring the relative abundance of SERINC3 and SERINC5 mRNA in human cells lines and primary rhesus macaque CD4+ T cells by qRT-PCR. (DOCX) [file ppat.1008487.s010.docx]

**S2 Table. Quantitative RT-PCR primers and probes for rhesus macaque *SERINC3* and *SERINC5* transcripts.**

| Primer/Probe | Sequence | |
| --- | --- | --- |
| Rh-SERINC3 Probe | 5’-/56-FAM/AGCATCCGC/ZEN/ACTTCCACTAATAGCC/3IABkFQ/-3’ | |
| Rh-SERINC3 Fw | 5’-CTGTTTGTCTTTGTTCTCTGCC-3’ | |
| Rh-SERINC3 Rv | 5’-CAGGGTCAGCTTGTCTACTTG-3’ |  |
| Rh-SERINC5 Probe | 5’-/56-FAM/AACAAGCTG/ZEN/TGGTACGCCTCCC/3IABkFQ/-3’ |  |
| Rh-SERINC5 Fw | 5’-AGTGGAACAAGAACTGGACAG-3’ |  |
| Rh-SERINC5 Rv | 5’-AACACTGCCATCAAAACCAAG-3’ |  |
| Rh-GAPDH Probe | 5’-/56-FAM/TCCCCACTG/ZEN/CCAACGTGTCA/3IABkFQ/-3’ |  |
| Rh-GAPDH Fw | 5’-GTCATCCCTGAGCTGAACG-3’ |  |
| Rh-GAPDH Rv | 5’-CCTGCTTCACCACCTTCTTG-3’ |  |
| Hu-SERINC3 Probe | 5’-/56-FAM/TGGACAACG/ZEN/AGAAAGAGGGAGTGC/3IABkFQ/-3’ |  |
| Hu-SERINC3 Fw | 5’-GTGGTGCCAGTGATGAAGAA-3’ |  |
| Hu-SERINC3 Rv | 5’-GCAGAGCATGAGGTGGAATAA-3’ |  |
| Hu-SERINC5 Probe | 5’-/56-FAM/TCCTGGTGG/ZEN/AGAGGACACTGAAGA/3IABkFQ/-3’ |  |
| Hu-SERINC5 Fw | 5’-GCAGCTCCTGAATTGGAGATAG-3’ |  |
| Hu-SERINC5 Rv | 5’-GGTGCCTTTCTTCTCGTCATAA-3’ |  |
| Hu-GAPDH Probe | 5’-/56-FAM/AGATCATCA/ZEN/GCAATGCCTCCTGCA/3IABkFQ/-3’ |  |
| Hu-GAPDH Fw | 5’-GAGTCCTTCCACGATACCAAAG-3’ |  |
| Hu-GAPDH Rv | 5’-GGTGTGAACCATGAGAAGTATGA-3’ |  |
